# Application of Inductively Coupled Plasma Spectrometric Techniques and Multivariate Statistical Analysis in the Hydrogeochemical Profiling of Caves—Case Study Cloșani, Romania

**DOI:** 10.3390/molecules26226788

**Published:** 2021-11-10

**Authors:** Anamaria Iulia Torok, Erika Andrea Levei, Silviu Constantin, Oana Teodora Moldovan, Marin Senila, Oana Cadar, Dorina Casoni, Simion Bogdan Angyus, Claudiu Tanaselia, Eniko Covaci, Tiberiu Frentiu

**Affiliations:** 1 INCDO—INOE 2000 National Institute for Research and Development of Optoelectronics, Research Institute for Analytical Instrumentation Subsidiary, Donath 67, 400293 Cluj-Napoca, Romania; iulia.torok@icia.ro (A.I.T.); erika.levei@icia.ro (E.A.L.); marin.senila@icia.ro (M.S.); oana.cadar@icia.ro (O.C.); bogdan.angyus@gmail.com (S.B.A.); claudiu.tanaselia@icia.ro (C.T.); 2 Department of Geospeleology and Paleontology, Emil Racovita Institute of Speleology, Calea 13 Septembrie, 050711 Bucharest, Romania; silviu.constantin@iser.ro; 3 Romanian Institute of Science and Technology, Saturn 24-26, 400504 Cluj-Napoca, Romania; oanamol35@gmail.com; 4 Centro Nacional de Investigación Sobre la Evolución Humana, Paseo Sierra de Atapuerca 3, 09002 Burgos, Spain; 5 Department of Cluj-Napoca, Emil Racovita Institute of Speleology, Clinicilor 5, 400006 Cluj-Napoca, Romania; 6 Department of Chemistry, Faculty of Chemistry and Chemical Engineering, Babes-Bolyai University, Arany Janos 11, 400028 Cluj-Napoca, Romania; dorina.casoni@ubbcluj.ro (D.C.); eniko.covaci@ubbcluj.ro (E.C.); 7 Research Center for Advanced Chemical Analysis, Instrumentation and Chemometrics-Analytica, Babes-Bolyai University, Arany Janos 11, 400028 Cluj-Napoca, Romania

**Keywords:** inductively coupled plasma optical emission spectrometry, inductively coupled plasma mass spectrometry, rare earth element, multivariate statistical analysis, principal component analysis, two-way joining analysis, hydrogeochemical profile, cave, water, soil

## Abstract

The aim of the study was to develop the hydrogeochemical profiling of caves based on the elemental composition of water and silty soil samples and a multivariate statistical analysis. Major and trace elements, including rare earths, were determined in the water and soil samples. The general characteristics of water, anions content, inorganic and organic carbon fractions and nitrogen species (NO_3_^−^ and NH_4_^+^) were also considered. The ANOVA—principal component analysis (PCA) and two-way joining analysis were applied on samples collected from Cloșani Cave, Romania. The ANOVA-PCA revealed that the hydrogeochemical characteristics of Ca^2+^-HCO_3_^−^ water facies were described by five factors, the strongest being associated with water-carbonate rock interactions and the occurrence of Ca, Mg and HCO_3_^−^ (43.4%). Although organic carbon fractions have a lower influence (20.1%) than inorganic ones on water characteristics, they are involved in the chemical processes of nitrogen and of the elements involved in redox processes (Fe, Mn, Cr and Sn). The seasonal variability of water characteristics, especially during the spring, was observed. The variability of silty soil samples was described by four principal components, the strongest influence being attributed to rare earth elements (52.2%). The ANOVA-PCA provided deeper information compared to Gibbs and Piper diagrams and the correlation analysis.

## 1. Introduction

Caves are nonrenewable geological formations that include mineral deposits; paleontological remains; distinctive microbiology and archaeological, historical and cultural material remains. Speleothems are secondary carbonate deposits formed in limestone caves by flowing or dripping water from the ceiling and walls of the cave to the floor. The formation of the speleothem is influenced by the concentration of the acids in the water, temperature and humidity of the cave; ground climate; annual rainfall and the density of the plant cover [1].

The seasonal monitoring of metals and metalloids of natural or anthropogenic origin in caves represents an important strategy to protect these unique resources. The major and trace elements, along with rare earth elements (REEs) and seasonal changes, could be considered as fingerprints of a specific cave and the identification of contamination sources. The solubility and bioavailability of metals depend on their oxidation state and affinity for the organic species. Iron and manganese oxides, as major components in the karst system, pH and redox conditions, influence the trace element chemistry and the equilibrium between their mobile and nonmobile species [2]. Soil bedrocks, cave deposits, detrital materials and sediments are natural sources of metals occurrences in water [3,4]. Elevated concentrations of Ca, Mg, Sr and K in a cave can be explained by mineral weathering of the local bedrock and soil atop the cave under the influence of water and their transfer to the cave through dripping [5].

Multivariate statistical techniques are frequently used to analyze the natural and anthropogenic origins of elements, their chemistry and water-rock/sediment interaction processes [6,7]. The principal component analysis (PCA) was used to identify the hydrogeochemical patterns of caves and to highlight the similarities and differences of the karst features [8]. The water characteristics in caves are influenced by water-rock interactions, recharge and discharge processes and anthropogenic activities in the area [9]. The mineral weathering may change the water salinity and element concentrations due to the dissolution at certain pH and redox conditions [7,10,11,12]. The dissolution/precipitation, hetero- or homogeneous acid/base reaction, phase transition, hydration/dehydration, microbial processes and redox reactions are the most common processes that can take place in a cave environment [13]. A Gibbs diagram can help to identify the processes that characterize the water-rock interaction, evaporation and precipitation, which may occur and influence the mineral contents and water types. The water types emerge from Ca^2+^-HCO_3_^−^ to Ca^2+^-Mg^2+^-HCO_3_^−^, Na^+^-HCO_3_^−^ or Na^+^-Cl^−^ [14].

The aim of this study was to apply inductively coupled plasma spectrometric techniques and a multivariate statistical analysis to the hydrogeochemical profiling of caves. A case study was conducted on Cloșani Cave, Romania, where water and silty soil samples from the cave floor were seasonally taken. The contents of the major elements were determined by inductively coupled plasma optical emission spectrometry (ICP-OES), and the contents of the minor elements, including REEs, were determined by inductively coupled plasma mass spectrometry (ICP-MS). The mineral compositions of the silty soil samples were investigated by X-ray diffraction (XRD). Along with the elementary profile of the water samples, the distribution on different inorganic and organic fractions of carbon (total carbon—TC, total organic carbon—TOC, total inorganic carbon—TIC, dissolved carbon—DC and dissolved inorganic carbon—DIC); the content of anions (HCO_3_^−^, F^−^, Cl^−^, NO_3_^−^, SO_4_^2−^ and PO_4_^3−^); NH_4_^+^; pH; electrical conductivity (EC); total dissolved substance (TDS) and alkalinity were determined. The chemical conceptual model, which described the pattern of hydrogeochemical processes in the cave considered as the case study, was conducted by a combination of classical Gibbs and Piper diagrams, the Stuyfzand Hydrogeochemical Classification System (SHCS) and unsupervised chemometric tools, such as ANOVA-PCA and heat maps obtained after a two-way joining analysis as a clustering method. This approach allowed a deep understanding of the hydrogeochemical processes that take place in a cave and underlie the characterization of the water and local geology. Additionally, a characterization of the seasonal variability of the physicochemical parameters of the water was performed. Such a complex approach of the hydrogeochemical processes in caves is of great interest to geochemists, speleologists and, last but not least, chemists.

## 2. Results

### 2.1. Hydrogeochemical Characteristics and Summary Statistics

The hydrogeochemical characteristics and summary statistics (minimum, maximum and median values; standard deviation and statistic parameters for distribution around the mean values) for the water samples seasonally collected from a small pool on the clayish floor (C2) and a small pond on calcite (C3) are presented in Table 1 and Table 2. The site descriptions, the sampling points and samples are presented in detail in Section 4.1.

### 2.2. Water Facies

**Figure 1:**
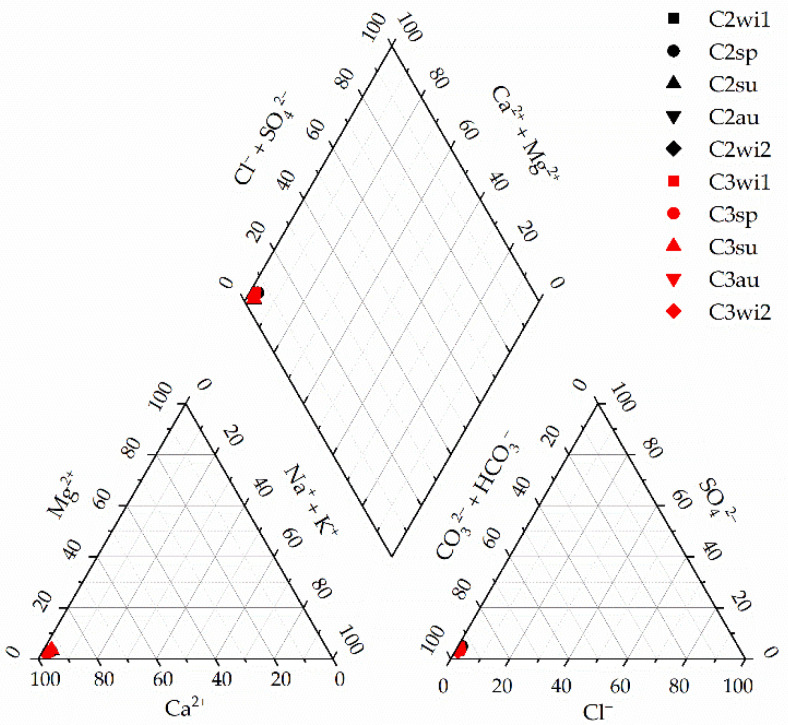
Piper trilinear diagram for the water samples collected from Cloșani Cave. C2—water samples collected from a small pool on the clayish floor; C3—water samples collected from a small pond on calcite. wi—winter; sp—spring; su—summer; au—autumn.

The hydrogeochemical facies of the water collected from Cloșani Cave according to the Piper trilinear diagram [15] are shown in Figure 1.

**Figure 2:**
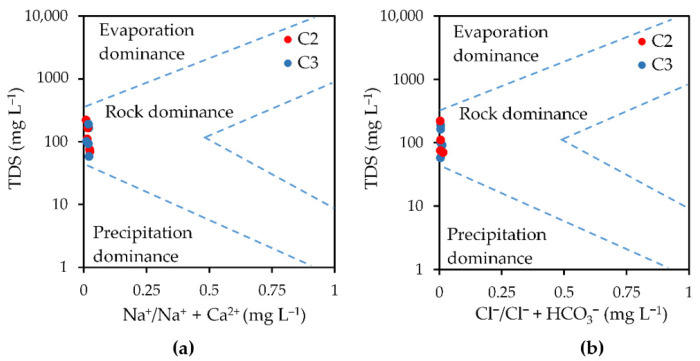
Gibbs diagram for Na^+^/Na^+^ + Ca^2+^ (**a**) and Cl^−^/Cl^−^ + HCO_3_^−^ (**b**) for the water samples collected from Cloșani Cave. C2—water samples collected from a small pool on the clayish floor; C3—water samples collected from a small pond on calcite.

The Gibbs diagram [16] for the cations (TDS vs. Na^+^/Na^+^ + Ca^2+^) and anions (TDS vs. Cl^−^/Cl^−^ + HCO_3_^−^) for the water samples collected from Cloșani Cave are shown in Figure 2a,b.

The relationships between the major ions in the water samples originating from Cloșani Cave, which revealed a possible source of the elements and seasonal influences for the two sampling points, are presented in Table 3 and Supplementary Materials Figures S1 and S2.

The elemental compositions for the five silty soil samples collected from site C1 are presented in Table 4. The powder X-ray diffraction results are presented in Table 5.

### 2.3. ANOVA-Principal Component Analysis

#### 2.3.1. ANOVA-Principal Component Analysis for Water Collected from Cloșani Cave

The principal components and eigenvalues, which describe the variances of the water parameters after normalization and Varimax rotation, are presented in Table 6.

**Figure 3:**
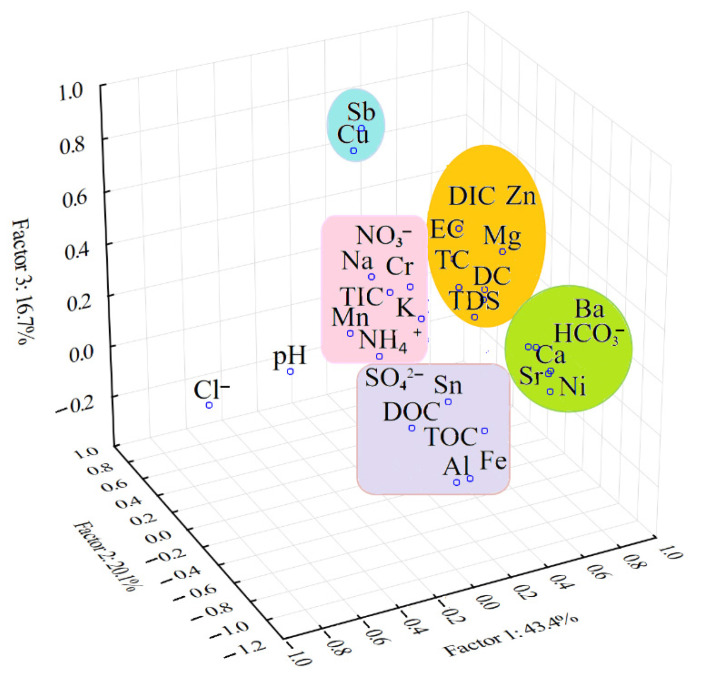
Three-dimensional Varimax-rotated PCA showing the grouping and interrelationships among the physicochemical characteristics of the water collected from Cloșani Cave.

Figure 3 presents the grouping of the water parameters by tridimensional Varimax-rotated PCA.

**Figure 4:**
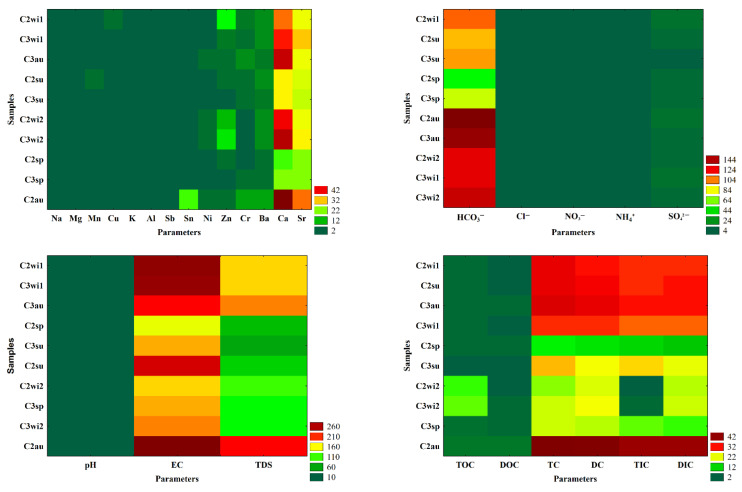
Heat maps after the two-way joining analysis as a clustering method to emphasize the seasonal variations, similarities or lack thereof between the water samples collected from Cloșani Cave. C2—water samples collected from a small pool on the clayish floor, C3—water samples collected from a small pond on calcite. wi—winter; sp—spring; su—summer; au—autumn. The measurement units of the parameters are presented in Table 1 and Table 2.

The patterns of the water samples collected from Cloșani Cave during different seasons and the similarities between the samples and characteristics or lack thereof could easily be observed in the heat maps obtained after the two-way joining analysis as a clustering method (Figure 4).

#### 2.3.2. ANOVA-Principal Component Analysis for Silty Soil Samples

Table 7 presents the principal components and eigenvalues, after the normalization and Varimax rotation, which describe the variances of silty soil samples in terms of the elemental compositions.

**Figure 5:**
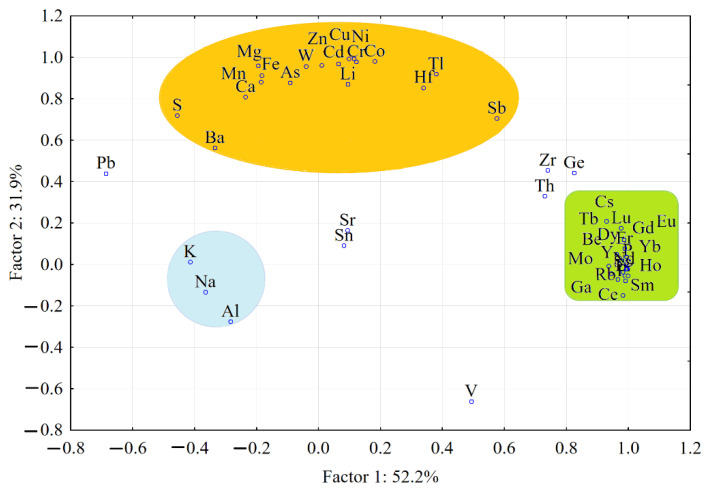
Two-dimensional Varimax-rotated PCA showing the grouping and interrelationships between the elements in the silty soil samples collected from Cloșani Cave.

**Figure 6:**
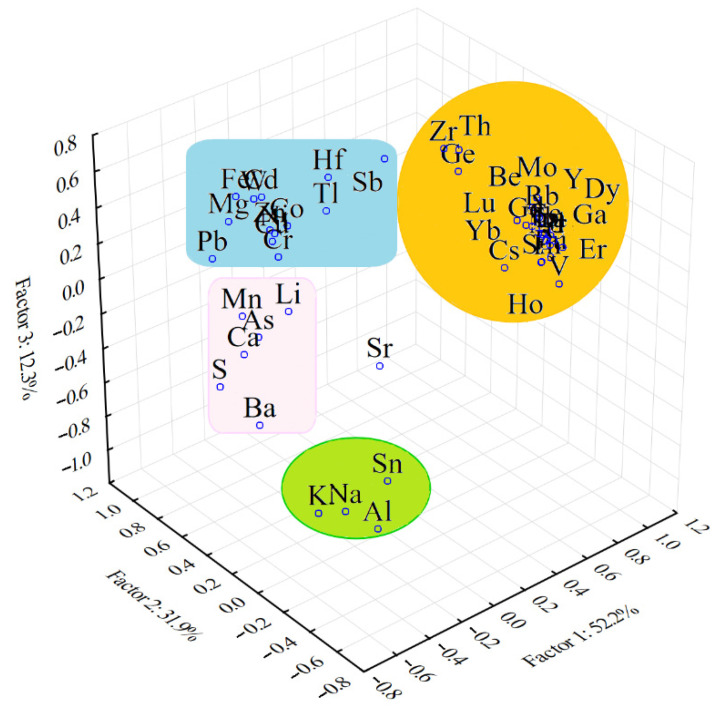
Three-dimensional Varimax-rotated PCA showing the grouping and interrelationships between the elements in the silty soil samples collected from Cloșani Cave.

The two-dimensional and three-dimensional Varimax-rotated PCA, presenting the grouping of the elements in the silty soil samples, are shown in Figure 5 and Figure 6.

**Figure 7:**
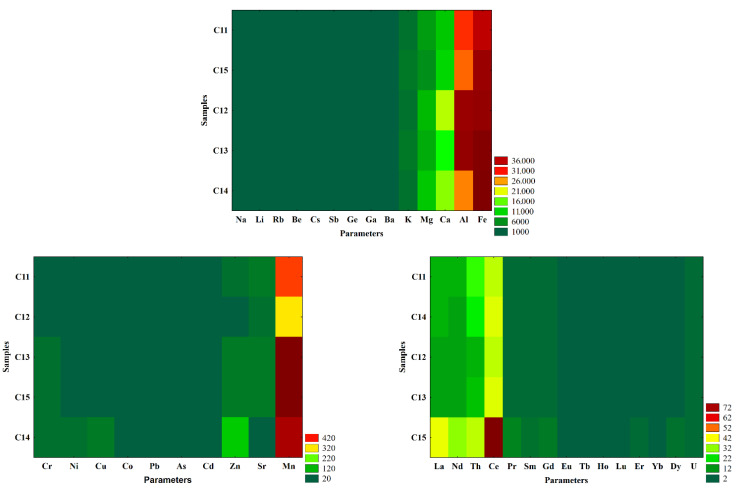
Heat maps after the two-way joining analysis as a clustering method to emphasize the variations of the elemental characteristics in the silty soil samples collected from Cloșani Cave. The measurement units of the parameters are presented in Table 3.

The pattern of the silty soil samples collected from Cloșani Cave and the similarities between the samples and characteristics or lack thereof could easily be observed in the heat maps obtained after the two-way joining analysis as a clustering method (Figure 7).

## 3. Discussions

### 3.1. Hydrogeochemical Modeling Based on Water Facies, Summary Statistics and Correlation Analysis

According to the data presented in Table 1 and Table 2, the pH of the water samples collected from Cloșani Cave was in the range of weakly acidic and weakly alkaline (6.6–8.2), which is consistent with the presence of a high concentration of Ca^2+^ (26.1–58.2 mg L^−1^) and HCO_3_^−^ (85–232 mg L^−1^) as a result of the water-rock interaction in the karstic system. The Ca^2+^ cation had a higher concentration of 50–100-fold than that of Na^+^, Mg^2+^ and K^+^. The concentrations of the toxic elements (Cd, Pb, Cr, Ni, Cu, Zn and As) and REEs were below the LODs in ICP-MS. The data presented in Table 1 and Table 2 showed that the water was characterized by a high bicarbonate concentration (85–232 mg L^−1^) and low concentrations for Cl^−^ (0.59–1.35 mg L^−1^), NO_3_^−^ (0.64–2.92 mg L^−1^), SO_4_^2−^ (3.6–10.5 mg L^−1^) and NH_4_^+^ (0.04–0.16 mg L^−1^), as a result of the water–carbonate rock interactions. In all the samples, the concentrations of PO_4_^3−^ and F^−^ were below the LOD in ionic chromatography. Generally, the increase of nitrite concentration in water is accompanied by a high concentration of sulfate under anthropogenic influence. Naturally, the waters seldom contained more than 5–10 mg L^−1^ of nitrate [6,18,19]. The pollution of water from Cloșani Cave by anthropogenic sources was low, because it did not surpass the threshold values (mg L^−1^: for NH_4_—0.5, Cl—250 and SO_4_^2^—250; μg L^−1^: for Cr—20, Cu—100, Zn—5000, Cd—5, Pb—10 and As—10) for groundwater bodies ROBA14 and ROJI02, respectively [20].

The concentrations (mg L^−1^) of TOC (1.2–16.6) and DOC (1.1–4.8) were very low in both sampling points compared to TIC (1.6–40.4) and DIC (10.1–40.1). In other words, the major carbon species in the water are in inorganic form (HCO_3_^−^). The *t*-test indicated that the physicochemical parameters in the water samples collected from the two sites were similar for the 95% confidence level (t_tab,υ1+υ2 = 8_ = 2.306), t_calc_ = 0.059–2.028, except for Cu (t_calc_ = 4.596) and NO_3_^−^ (t_calc_ = 2.311). In the case of Cu, the significant difference could be explained by the fact that, in some samples, its concentration was below the LOD in ICP-MS. In the case of NO_3_^−^, the significant difference was in agreement with the greatest differences observed between the samples collected in the two sites during the autumn and summer. The pH and alkalinity showed clear seasonal patterns in values that could be related to the dynamic changes in the environment. Therefore, the pH became weakly acidic during the summer and autumn compared to the weakly basic character during the spring and winter. It is known that the precipitation is the most acidic during the growing season (summer) and least acidic in the winter, when the hydrogen ion concentration in snowpack is lower [21]. Additionally, in the case of caves, the partial pressure of CO_2_ at the water-atmosphere interface is responsible for the water pH value and HCO_3_^−^ content. During the spring and summer, CO_2_ is significantly undersaturated. At the same time, the biological activity could be responsible for the water pH and alkalinity, but this behavior is negligible for Cloșani Cave, as shown by the very low values of TOC and DOC compared to TIC and DIC. Generally, the environment in caves is conservative, but this one is under the influence of nonconservative external processes. Therefore, a possible explanation for the pH and alkalinity pattern could be related to the infiltration of melted snow and runoffs. The pH and CO_2_ presence determined severe changes regarding the mineral content of the water—namely, Ca^2+^, HCO_3_^−^ and TDS.

The data in Table 1 and Table 2 demonstrate an asymmetric distribution of the parameters around the mean, except for EC, TC, DC and NH_4_^+^ in the case of the water collected from the C2 and C3 sampling points. The parameters showed either a positive or negative skewness. Some of them (Al, Cr, Sb, TOC, DOC, Cl^−^, SO_4_^2−^ and NH_4_^+^) showed leptokurtic distribution (kurtosis > 3) caused by some significantly different outliers, while Ba showed a leptokurtic and platykurtic (kurtosis < −3) distribution in the C2 and C3 sampling points, respectively. Consequently, the median values for these parameters were more relevant for the water characterization.

The water-rock interactions, such as the dissolution/precipitation of the minerals, composition of recharged water, evaporation, mixing processes and anthropic activities, are processes that control the chemical composition of the water in karstic rocks. According to the Piper trilinear diagram (Figure 1), it can be seen that the water samples collected from Cloșani Cave showed Ca^2+^-HCO_3_^−^ facies, typical for a karstic aquifer. The results are in agreement with the rock characteristics rich in Ca, Mg, Na and K that result in a weakly alkaline or acidic pH of water following rock leaching.

The Gibbs diagram (Figure 2a,b) revealed that the dominant natural process that controls the water chemistry is the water-rock interactions. A leaching study performed on silty soil samples provided a pH of leachate in the range 7.9–8.6. The results suggest that the water-rock interactions, namely the dissolution of Ca and Mg carbonates by water containing CO_2_ and desorption/adsorption processes from/on silicate rocks, could represent the main pathways resulting in an elevated content of Ca and bicarbonate in the water. According to relationships between the major ions in the water samples originating from Cloșani Cave presented in Table 3 and Supplementary Materials Figures S1 and S2, a seasonal influence on the water from the two sampling points could be observed. At the same time, the possible origin of the major components could be evaluated. The weak correlation between Na^+^ and Cl^−^ indicated the fact that the halite dissolution from rock was not the principal source of Na. The Na^+^:Cl^−^ ratio (mEq L^−1^) in the majority of the samples lying above the 1:1 line corresponded to the NaCl stoichiometric ratio. Similarly, there was no correlation between Na^+^-HCO_3_^−^ and Na^+^-SO_4_^2−^, all the samples being below the 1:1 stoichiometric ratio. Therefore, the dissolution of the other minerals, such as Na-bearing silicates, and the cation exchange between Na^+^ and Ca^2+^/Mg^2+^ on clays may act as the Na source [22]. The results are similar for K^+^, as there was no correlation versus Cl^−^, HCO_3_^−^ and SO_4_^2−^, the ratios being very different from the stoichiometric 1:1 line. With respect to Ca^2+^ and Mg^2+^, the determination coefficients (0.6250, 0.6148, 0.5248 and 0.5577) indicate that the weathering of Ca and Mg from carbonates by water in the presence of CO_2_ and sulfate minerals are the principal sources for the occurrence of Ca^2+^ and Mg^2+^ in water [10,11,23]. A seasonal influence, mainly during the spring, on the processes of the occurrence of major ions in the water could be observed by the results presented in Table 3 and Supplementary Materials Figures S1 and S2. Thus, a strong influence on the Na^+^-Cl^−^, Na^+^-HCO_3_^−^, Ca^2+^-HCO_3_^−^ and Mg^2+^-SO_4_^2−^ correlations could be observed. However, the processes of the water-rock interactions and the influence of external conditions on the occurrence of major ions in water and its characteristics are difficult to solve by a simple statistical approach given the complexity of the conditions in a cave. Obviously, a much more thorough approach, such as unsupervised statistical techniques, to obtain the relevant model as proposed in this paper is needed. 

The Stuyfzand Hydrogeochemical Classification System (SHCS) [24] defined the water samples as a very oligohaline main type (G), according to the Cl^−^ concentration < 5 mg L^−1^, a moderately low alkaline type (1) during the spring for both sampling sites and a moderate alkaline type (2) in the other seasons according to the HCO_3_^−^ concentration in the range 1.4–3.8 mEq L^−1^, the Ca^2+^-HCO_3_^−^ subtype (all samples) according to the dominant cation and anion and, finally, class (+) according to the salinity, which gave a (Na^+^+K^+^+Mg^2+^) surplus, often indicative of a fresh water intrusion anywhere and anytime. The results were similar to those discussed previously, regarding the influence of the season on the correlations between the main ions and the characteristics of the water from Cloșani Cave.

The data presented in Table 4 showed that Ca was the main element in the silty soil samples, followed by K, Mg, P, Na, Al and S. Most of the elements had mean concentrations below 40 mg kg^−1^, except for Fe, Mn, Ba, Zn and Sr. The mean concentrations of the REEs were in the range of 0.45 mg kg^−1^ (Tb)–44.6 mg kg^−1^ (Ce). Lanthanum and Nd had mean concentrations of 19.2 mg kg^−1^ and 17.4 mg kg^−1^, respectively. Additionally, the data presented in Table 4 showed a symmetric distribution for the Ca, V, Cu, Zn, Ba and Sr concentrations, while the other elements, including the REEs, had an asymmetric one. Phosphorus, Be, Sc, Ru, Y, Sn, W, Ga and the REEs, except for Hf, showed a leptokurtic distribution, while Al and Sb had a platykurtic one. Consequently, the median values for the concentrations of these elements were more relevant for the silty soil sample characterizations.

The XRD analysis (Table 5) revealed the presence of quartz (all samples) and sodium aluminosilicate (two samples) as the major minerals (20–50%). Calcite (all samples); minerals from the silicates group, such as muscovite calcian (all samples); Na, K, Ca, Mg and Fe aluminosilicate and albite (two samples) were identified as minor-level elements (5–20%). Other K and Ca aluminosilicates, such as illite, microcline, gismondine and gypsum, were identified as trace constituents in all the samples. The contents of the major elements in the silty soil samples determined by ICP-OES are in agreement with the XRD results.

### 3.2. Modeling by ANOVA-Principal Component Analysis

#### 3.2.1. ANOVA-Principal Component Analysis Applied for Water Collected from Cloșani Cave

The five principal components described almost 92% of the total variance of the water parameters after normalization and Varimax rotation (Table 6). The first factor (F1), accounting for 43.4% of the total variance, was associated with the water-carbonate rock interactions and dissolution of Ca and Mg from the host rocks in the presence of CO_2_. The Ca:Mg ratio in karst water is an important indicator of the geochemistry of the phreatic karst system. The Ca:Mg ratio can be connected to the proportion of calcite and dolomite from the aquifer rock and can be used as a qualitative indicator of the geochemical evolution of aquifers [25]. A high Ca:Mg ratio in water is explained by the flow of water through limestones, while a lower one is the result of the presence of other magnesium-based rocks in addition to carbonates, as shown by the XRD analysis in the case of Cloșani Cave. However, the strong loadings of Mg^2+^, Ca^2+^ and HCO_3_^−^ and the weak loadings of SO_4_^2−^ indicated that the main source of these two elements were carbonate rocks and not sulfate ones. The main process of ion occurrence in the water could not be highlighted by the correlations Ca^2+^-HCO_3_^−^, Ca^2+^-SO_4_^2−^, Mg^2+^-HCO_3_^−^ and Mg^2+^-SO_4_^2−^, while this was substantially clarified by the PCA approach. Thus, the PCA indicated that sulfate rocks had little influence on the variability of the Cloșani Cave water characteristics, as indicated by the loading factor values for SO_4_^2−^ in those five PCs. The release of H_2_S into the cave atmosphere was negligible in the case of oxygen-rich bicarbonate water, because it induced the oxidation to sulfate [26]. Several geogenic elements—namely, Ni, Sr and Ba—have a strong influence on water and originate from carbonate rocks. It is known that the Ba^2+^ ions are almost absent in infiltration water. Under these conditions, the water is oversaturated with barium and calcium sulfate, respectively. At near-neutral pH conditions similar to those found in Cloșani Cave, the presence of Ba in water even in a low concentration enhances the dissolution rate and solubility of quartz as much as forty times as compared to deionized water, thus having a strong influence on the mobilization of trace elements from rocks [27]. Most trace elements have a weak influence on the water characteristics, except for Cr, which has a moderate one. This demonstrates that the trace elements do not originate from carbonate rocks.

The total dissolved substance is positively correlated with the concentrations of major cations and anions (HCO_3_^−^) in water. The negative correlation between the cations and Cl^−^ indicate that the presence of Cl^−^ in water inhibits the leaching of cations from host rocks. 

A weak influence of TOC and DOC on the water characterizations from Cloșani Cave could be observed in this factor compared to DIC and TC, which had strong influences. The results are in agreement with Ca^2+^-HCO_3_^−^ water facies. However, the microbial films play an important role in the formation of barium-containing deposits in catacombs and have a direct role in the biotic control of silica precipitation [28,29].

The decrease of pH (a moderate influence in Factor 1) favors geogenic element dissolution [30]. Galdenzi [31] showed that the slow water-rock interaction in the presence of sulfate is considered to be the main cave-forming process and is associated with the release of HCO_3_^−^ ions from limestone dissolution. Therefore, the positive loadings of Ca and HCO_3_^−^ are consistent with the water-calcite interaction. Additionally, it is known that the organic matter plays a key role in the mobilization of trace elements precipitated by the calcium carbonate in soil [32]. Organic materials increase the release of metal species mainly by the formation of metal organic complexes [33]. Several elements, such as Mg, Ca and Ba, are essential for the microbial metabolism/growth in caves [29]. The metal mobilizations from the host rock have been described in hypogenic caves in limestone [34,35] and in peculiar iron-silica caves in Brazil [36].

The second factor (F2) describing 20.1% of the total variance was associated with inorganic and organic carbon chemistry (TIC and TOC) and the elements involved in redox processes (Fe and Al) whose oxidation state was influenced by the reducing/oxidizing conditions. The weak loading of DOC in this factor was in agreement with the oxidation of organic carbon, as shown in the reverse strong correlation between TOC and TIC. The second factor was also associated with Fe cycling, possibly by the presence of iron-reducing microorganisms that could mobilize insoluble Fe(III) from host rocks into soluble Fe(II) in water. On the other hand, although there was a low concentration of organic carbon in the water from Cloșani Cave, this is sufficient to mobilize Fe following the reduction process by microbial activity. Therefore, the presence of organic carbon suggests that microbial activity occurs within Cloșani Cave. The mobilization of Fe by microbial activity was inhibited by the presence of Cl^−^ in the water.

The third factor (F3; 16.7% variability) was associated with a strong influence of some trace elements (Cu, Zn and Sb).

The fourth factor (F4; 6.7% variability) was associated with the inorganic nitrogen chemistry in the karst aquifer within Cloșani Cave, highlighted by the strong loading factors of NO_3_^−^ and NH_4_^+^ ions and some trace elements, such as Sn (strong influence) and Cr (moderate influence). These elements can be involved in redox processes suffered by nitrogen species. Water naturally seldom contains more than 5–10 mg L^−1^ of NO_3_^−^, as in the case of the samples collected from Cloșani Cave. Cuoco et al. [18] showed that any deviation from the natural character of the groundwater in the HCO_3_^−^-Cl^−^-SO_4_^2−^ system by an increase in the sulfate of anthropogenic origin was accompanied by an increase in the nitrate content. As the nitrate and sulfate concentrations were low in the water samples collected from Cloșani Cave, it can be concluded that their origin was due to natural processes, under microbial activity, even in the presence of a low organic matter content. Thus, the presence of nitrate in the water from Cloșani Cave could be the result of the nitrification of organic matter with nitrogen on sedimentary rocks, followed by the oxidation of organic matter in the presence of bacteria and subsequent leaching by water. The nitrate anion acts as an electron acceptor in the oxidation of organic matter under anoxic conditions. This process was highlighted by the strong loading of DOC in this factor and a positive correlation with inorganic nitrogen species. Therefore, the nitrate concentration in the water was determined by the available concentration of organic matter deposited or embedded in the sedimentary host rocks. Ammonium ion was the final product of the microbial reduction of nitrate. Since there was a positive correlation between the two nitrogen species, they had a common source through the processes previously described. In other words, the presences of nitrate and ammonium depend on the conditions within a cave aquatic environment. Supplementary discussions of nitrate origins in karst aquifers and earth caves are presented in the literature [37].

The fifth factor (F5; 4.7% variability) was associated with the presence of K and Mn (a strong, positive influence) and the hydrogeochemical processes of their occurrence in water. The strong Mn-K correlation suggests that the occurrence of K in the water is due to the leaching of manganese-based rocks. On the other hand, although there was a low concentration of organic carbon in the water, the coming inflow into the Cloșani Cave system is sufficient to mobilize K and Mn following the reduction process by microbial activity. Therefore, the presence of organic carbon in water suggests that a microbial activity takes place within Cloșani Cave.

In the tridimensional Varimax-rotated PCA (Figure 3), three important groups were very well-separated, namely: (i) the group of inorganic carbon (HCO_3_^−^) highly correlated with several elements (Ca, Ba, Sr and Ni) that have a strong influence on the water characteristics; (ii) the group that comprises the dissolved carbon species (DC and DIC), TC, the minerals containing Zn and Mg and general characteristics of water (TDS and EC) and (iii) the group containing nitrogen species; organic carbon fractions (TOC and DOC) and the elements Al, Mn, Fe, Na, K and Sn. Two trace elements (Sb and Cu) very strongly correlated between them were not included in any of the groups, as they had very low concentrations in some water samples and were below the LOD in ICP-MS. A lack of correlation between the inorganic and organic carbon species in water could also be observed. There was also a weak correlation of pH and Cl^−^ versus the parameters that described the physicochemical characteristics of the water from Cloșani Cave. This observation was in agreement with the fact that the water pH did not present a strong influence in either PC obtained after the Varimax rotation (Table 6). Additionally, as shown previously, there was a significant seasonal variability of the water pH.

The pattern of the water samples from Cloșani Cave collected in different seasons (Figure 4) was described mainly by the following elements: Ca, Al, Fe, Ba and Sr; inorganic carbon species or fractions (HCO_3_^−^, TC, DC, TIC and DIC); TDS and EC. Additionally, the two-way joining analysis as a clustering method emphasized that the carbon organic fractions were not correlated with those of inorganic carbon, and their values were not substantially influenced by the season and did not provide fingerprints in the seasonal discrimination of the water samples. These observations were consistent with the outcomes in the Varimax rotation, which showed that the mineral components had the greatest influence on the variability of the water properties.

#### 3.2.2. ANOVA-Principal Component Analysis Applied for Silty Soil Samples Collected from Cloșani Cave

The results presented in Table 7 show that four principal components described 100% of the total variance of the elemental composition of the silty soil samples after normalization and Varimax rotation.

The first factor that described 52.2% of the variability was associated with the REEs, which had a strong influence on the characteristics of the silty soil samples. In this factor, there P was also included, and the following trace elements with a strong influence: Be, Sc, Rb, Y, Zr, Mo, Cs, Th and Ga were strongly positively correlated with the REEs. The presence of lanthanides is usually also reported in other cave environments [38,39,40]. The crystallographic parameters, such as the ionic radius, amount of crystal defect sites and adsorption potential of the rock surfaces, may have a direct effect on the embedding of the REEs and other elements, such as Be, Sc, Rb, Y, Zr, Mo, Cs, Th and Ga, in P-based rocks. An increase in the pH of water similar to Cloșani Cave resulted in the increasing of REE adsorption onto the surfaces of minerals and their precipitation. This explains the very low concentration of REEs in the water and their presence in the solid phase. The patterns of the REEs (concentration in deposits, fractionation into light and heavy REEs and ratio of certain isotopes, such as those of Nd) of the Y and the elements correlated with them in the solid phase could be closely related with climate changes and the cave environment in particular and could be considered as a useful tool to approach paleochemistry studies in caves [38,41]. A supplementary study would be needed in the case of Cloșani Cave.

The second factor that described 31.8% of the variability was associated with Mg, Ca, Fe and Ba rocks, which imbedded S and several trace elements, mainly Cr, Mn, Co, Ni, Cu, Zn, As, Cd, Sb, W, Tl and Hf. Antimony and thallium are closely associated in carbon-rich rocks. The positive correlation with Fe and Mn indicated that the rocks containing Fe-Mn oxides are the host of heavy trace elements. It is known that natural deposits contain oxides of Fe-Cu-Zn-Pb, which incorporate traces of Cd, Co, Ni, etc. [42,43].

The third factor (12.3% from the total variability) was associated with Na, K, Al, Sn and Ba-based rocks. The lack of correlation with Ca and S led to the idea that gypsum-rich evaporites are not the hosts of these elements.

The last factor that described only 3.6% of the variability was associated with strontium-based rocks, which could also incorporate V. However, the influence of V was not clearly highlighted on the elemental composition variability, because this element had a weak influence (Factors 1, 3 and 4) and moderate one (Factor F2).

Figure 5 and Figure 6 show the grouping of REEs very strongly correlated between them that demonstrates a low variability and a strong influence on the characteristics of the silty soil samples collected from Cloșani Cave. The group of trace elements show a high variability in the analyzed samples. Indeed, Figure 6 indicates a weak correlation of V with the other trace elements and REEs and a weak influence on the elemental composition variability of the silty soil samples. The group of aluminosilicates shows a very strong correlation between Na, K and Al, which embeds a few trace elements, such as Sn.

It could be observed in Figure 7 that there were no evident variations of the trace elements and REEs in the silty soil samples except for Mn, La, Nd, Th and Ce. The variations of major element contents (Ca, Mg and Al) could indicate that the precipitation and growing of calcite and silicate could be linked with variations of La, Nd and Ce.

## 4. Materials and Methods

### 4.1. Site Description, Sample Collection and Preservation

Cloșani Cave is located in Southwestern Romania at 433 m a.s.l. It is one of the most famous and studied caves, as it hosts a laboratory for biospeleological, crystallogenetic and topoclimatic studies [44,45,46]. It is a protected cave with two major passages summing about 1.5 km abounding in a wide range of spectacular speleothems. The cave was developed from Upper Jurassic-Aptian limestones and is characterized by a constant temperature around of 11 °C, a relative air humidity of 99–100% and a pCO_2_ value (ppm) of 8800 in the summer and 1500 in the winter. The cave hosts about 16 species of cave endemics, with a very limited distribution [47].

**Figure 8:**
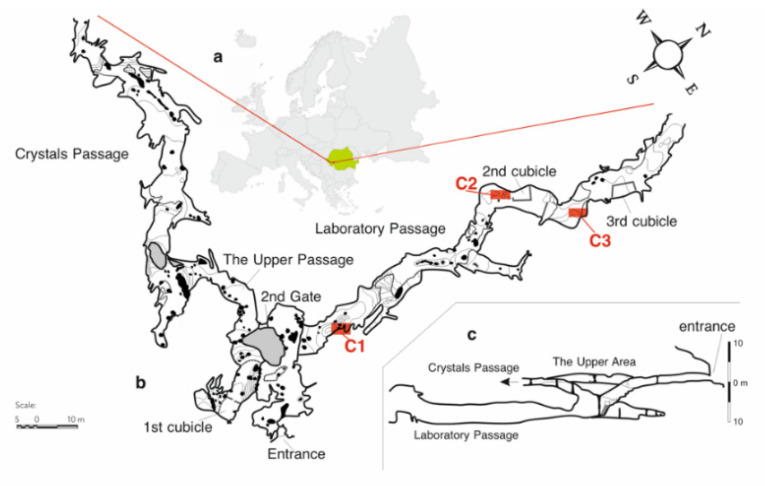
The location of Cloșani Cave in the southwestern part of Romania (**a**). The sampling points (C1—silty soil samples and C2 and C3—pool water samples) in the laboratory passage (**b**). The profile of the cave (**c**).

Ten water samples (aliquot volumes of 3 L) were collected, five each from two sampling points located in the laboratory passage (C2 and C3) in the winter (February, wi1), spring (May, sp), summer (August, su) and autumn (November, au) 2019 and winter (February, wi2) 2020 in precleaned polyethylene bottles. Water samples were kept at 4 °C until analysis, when they were divided into three replicates and prepared for analysis according to Section 4.3.1. Collection point C2 was a small pool on the clayish floor, and C3 was a small pond on calcite, both fed by drip water. In the same sampling campaigns, five silty soil samples (about 100 g) were collected from the cave floor (C1 sampling point) in plastic bags closed and kept at 4 °C until analysis. The sampling points are shown in Figure 8.

### 4.2. Reagents and Certified Reference Materials 

Stock solutions of 1000 mg L^−1^ of F^−^, Cl^−^, NO_3_^−^, SO_4_^2^^−^ and PO_4_^3^^−^; 1000 mg L^−1^ of potassium hydrogen phthalate; 1000 mg L^−1^ of NH_4_Cl; Na_2_CO_3_ 99.6% (*w*/*w*); NaHCO_3_ 99.9% (*w*/*w*); 30% (*w*/*w*) ultrapure hydrochloric acid and 60% (*w*/*w*) ultrapure nitric acid were purchased from Merck, Darmstadt, Germany. Multielement Calibration Standard 2 containing 17 REEs of 10 mg L^−1^ in 5% HNO_3_; Multielement Calibration Standard 3 containing 29 elements of 100 mg L^−1^ in 5% HNO_3_ and Instrument Calibration Standard 3 containing Ca, Fe, K, Mg and Na 1000 mg L^−1^ in 5% HNO_3_ were purchased from Perkin-Elmer, Waltham, MA, USA. Calibration standard solutions were prepared by serial dilution. Ultrapure water (18 MΩ cm) prepared in the laboratory with PureLab flex, Elga Veolia, High Wycombe, UK, was used throughout the study.

Certified reference materials (CRMs), such as NIST SRM 1643f Trace Elements in water, National Institute of Standards and Technology, Gaithersburg, MD, USA, METRANAL 32 Light Sandy Soil Elevated Analyte Levels, Analytika, Prague, Czech Republic, CRM 048-50G Trace Metals—Sand 1 and LRAC6625 Loamy Clay, Sigma Aldrich RTC, Laramy, WY, USA, NCS ZC 73,006 Soil and NCS DC 78,301 River Sediment, China National Analysis Center for Iron and Steel, Beijing, China, GBW 07,404 Soil, Institute of Geophysical and Geochemical Exploration, Langfang, China and BCR—280R Lake Sediment, Institute for Reference Materials and Measurements—IRMM, Geel, Belgium, were used to check the accuracy of ICP-OES and ICP-MS methods for the determination of the element concentrations. The CRM QC1308-drinking water, Sigma Aldrich RTC, Laramy, WY, USA, was used to validate the determination of the TOC and DOC fractions. Certified SPS-NUTRWW1 wastewater, Spectrapure Standards, Oslo, Norway, was used to validate the chromatographic method for anion determination.

### 4.3. Sample Preparation and Chemical Analysis

#### 4.3.1. Water Samples

An aliquot volume of 1 L from collected water samples was filtered through 0.45-μm cellulose acetate membrane filters and acidulated with 2% (*v*/*v*) ultrapure HNO_3_ for element determination by ICP-OES and ICP-MS. Dissolved carbon (DC) and dissolved inorganic carbon (DIC) were determined in filtered samples, while the total carbon (TC) and total inorganic carbon (TIC) were determined in the original water samples without filtration using a 2100S Multi N/C analyzer, Analytik Jena (Jena, Germany), based on nondispersive infrared absorption measurements of CO_2_. For the TC and DC measurements, an aliquot volume of 500 μL of sample was injected into the combustion furnace, thermocatalytically pyrolyzed and oxidized at 950 °C in the presence of a Pt/Al_2_O_3_ catalyzer, followed by cooling, drying and dehalogenation prior to measurements. For the TIC and DIC measurements, the CO_2_ was generated from an aliquot volume of 500 μL of sample injected into the inorganic carbon reactor in the presence of 1 mL of 10% (*w*/*w*) H_3_PO_4_. Total organic carbon (TOC) and dissolved organic carbon (DOC) was determined by the differences between TC and DC and TIC and DIC, respectively. The concentrations of the anions (Cl^−^, F^−^, SO_4_^2−^, NO_3_^−^ and PO_4_^3−^) were determined by ion chromatography in filtered and non-acidulated water samples in a Na_2_CO_3_-NaHCO_3_ buffer solution as the mobile phase at a flow rate of 0.7 mL min^−1^ using a 761 Compact IC Metrohm ion chromatograph, Metrohm, Herisau, Switzerland, equipped with a chemical suppression module and conductivity detector. The total water alkalinity, expressed as mg L^−1^ HCO_3_^−^, was quantified by the titration of 100 mL of filtrated aliquot sample with 0.1 mol L^−1^ of HCl in the presence of methyl orange. The concentration of NH_4_^+^ ions in the filtrated water was determined by Vis molecular absorption using the salicylate method. In the first stage, the ammonium ion was reduced by the hypochlorite anion to monochloramine, followed by a reaction with the salicylate anion to form 5-aminosalicylate at pH 12.6 and the catalytic oxidation of the product in the presence of sodium nitroso pentacyanoferrate (III). The absorbance of the green-colored solution was measured at 650 nm using a Lambda 25 spectrophotometer from Perkin Elmer, Waltham, MA, USA. Hypochlorite anions were generated in situ by the alkaline hydrolysis of the sodium salt of N, N-dichloro-1,3,5-triazine-2,4,6 (1H, 3H and 5H)-trione. Sodium citrate was added to mask the interference of the calcium and magnesium cations. The pH and EC were measured using the Seven Excellence multiparameter Mettler Toledo, Greifensee, Switzerland. The TDS content was determined after evaporation at 105 °C of an aliquot of 100 mL of filtered water. These procedures were described by Eaton and Greenberg [48]. Three parallel measurements (*n* = 3) were performed for each parameter.

#### 4.3.2. Soil Samples

The silty soil samples collected from the cave floor were dried at 110 °C for 24 h, ground to a fine powder and sieved (<150 μm). Amounts of 3 g of powder in triplicate were digested in 28 mL of a 3:1 (*v*/*v*) HCl (30%):HNO_3_ (60%) mixture in a sand bath, made up to 100 mL and then filtrated. The contents of the elements in the digests were determined by the ICP-OES and ICP-MS methods. A mineralogical analysis was carried out on powder-pressed pellets by X-ray diffraction (XRD) using the D8 Advance Diffractometer, Bruker, Karlsruhe, Germany, at CuK_α_ radiation (λ = 1.54060 Å), 40 kV operating voltage and 40 mA current.

### 4.4. Method Validation

The concentrations of Al, Na, Mg, Ca, K, P and S (axial viewing) and Fe (radial viewing) in the water and digested samples were determined by ICP-OES using a 5300 Optima DV spectrometer, Perkin Elmer, Waltham, MA, USA, equipped with a pneumatic nebulizer. The concentrations of Li, Be, Sc, V, Cr, Mn, Co, Ni, Cu, Zn, As, Rb, Sr, Y, Zr, Mo, Cd, Pb, Sn, Sb, Ba, Cs and the REEs were determined by ICP-MS using Elan DRC II from Perkin Elmer, Waltham, MA, USA. The operating conditions for the ICP-OES and ICP-MS instruments are presented in Supplementary Materials Tables S1 and S2. Most of the trace elements were determined by ICP-MS in the standard operation mode without DRC and the isotopic internal standards. Some of the elements (Mo, Ba, Nd, Sm, Gd, Dy and Yb) were determined without DRC but using the default mathematical modeling isobaric interference for the corresponding isotopes. The determination of As was carried out by a polyatomic ion (^75^As^16^O)^+^ using DRC mode (RPq = 0.45, O_2_ reaction gas 0.4 mL min^−^^1^) in order to avoid the polyatomic ^40^Ar^35^Cl^+^ interference on the ^75^As isotope.

The spectrometric methods were characterized in terms of the limit of detection (LOD), linearity of calibration curves, precision and recovery. The limit of detection was estimated using the (3σ) criterion and parameters of the calibration curve [49].

(1) $$\mathrm{LOD}=\frac{3{\;s}_{b}}{m}$$

where (s_b_) is the standard deviation of the background assessed from 11 measurements of the reagent blank (2% *v*/*v* HNO_3_), and (m) is the slope of the calibration curve.

The LODs in the silty soil samples were calculated by taking into account the LODs in the liquid and the sample preparation protocol (3 g of sample digested and made up to 100 mL). The linear calibration function was evaluated according to SR ISO 8466-1 and Covaci et al. [50,51]. The accuracy of the ICP-OES and ICP-MS methods was assessed through a recovery assay by comparing the found results with certified values of elements in the water, sediment and soil CRMs for the 95% confidence level (*n* = 3 parallel measurements). A pooled recovery for each element was calculated based on the results obtained from the analysis of the CRM samples. Precision was evaluated as the internal standard deviation of repeatability (s_r_, %) obtained in the analysis of the real samples (*n* = 3) [51,52]. The LODs for ICP-OES and ICP-MS are summarized in Supplementary Materials Tables S3 and S4. 

The LODs in ICP-OES were in the range of 3 (K)–50 (P and S) μg L^−1^ in the water and 0.10 (K)–1.67 (P and S) mg kg^−1^ in the silty soil samples. In ICP-MS, the LODs were in the range of 0.013 (Cr)–0.61 (Cs) μg L^−1^ in the water and 0.007 (Cr)–0.020 (Cs) mg kg^−1^ in the silty soil samples. In the case of the REEs, the LODs in ICP-MS were in the range of 0.022 (Lu and Hf)–0.053 (Gd) μg L^−1^ in the water and 0.0008 (Lu and Hf)–0.0018 (Gd) mg kg^−1^ in the silty soil samples.

The results obtained in the analysis of the CRMs by ICP-OES and ICP-MS are presented in Supplementary Materials Tables S5–S8. The ICP-OES method was validated for multielemental determination in the water, with recovery in the range of 96–104% and trueness in the range of ±3 to ±5%, while ICP-MS was with recovery in the range of 88–113% and trueness in the range of ±3 to ±5%. The recovery for multielemental determination by ICP-OES in the solid samples was in the range of 95–101% and trueness in the range of ±7 to ±12%. The ICP-MS method was validated for the multielemental determination of the common elements in solid samples, with recovery in the range of 91–104%, and trueness in the range of ±5 to ±16%. A recovery of 81–101% and trueness of ±4 to ±9% were obtained for ICP-MS in the determination of the REEs in the soil samples. The precision, expressed as the relative standard deviation (RSD) of both spectrometric methods (*n* = 3 parallel measurements for each sample), was better than 10%.

The operating conditions for the 761 Compact IC Metrohm ion chromatograph, Herisau, Switzerland, used for the determination of the anion contents in the water samples are provided in Supplementary Materials Table S9, while the LODs are in Table S10. Ion chromatography provided recovery, trueness and precision as RSD in the range of 95–107%, ±5 to ±8% and 3.3–7.7% (*n* = 3 parallel measurements for each sample), respectively [6]. The detection limits for the anions in water samples were in the range of 0.004(Cl^−^)–0.100 NO_3_^−^ mg L^−1^.

The spectrophotometric method used for ammonium determination in the water samples was validated with a detection limit of 0.01 mg L^−1^, a mean recovery of 98% and a trueness of ±4%, while the RSD (*n* = 3 parallel measurements for each sample) was better than 14%. The precision for the determination of the electrical conductivity in the water samples was better than 4%, while the LOD was 1.09 μs cm^−1^. The determination of the total organic carbon was validated, with a recovery of 103 ± 4% and a precision better than 6%. The detection limits of the carbon fractions in the water (mg L^−1^) were 0.30 (TOC, TIC and TC) and 0.40 (DOC, DIC and DC). The determination of the alkalinity was validated with a precision in the range of 2.9–6.4% and a LOD of 20 mg L^−1^ HCO_3_^−^. The LOD for TDS was 3.0 mg L^−1^. The water pH was determined with a precision better than 5% and an accuracy of ±0.1 pH units.

### 4.5. Multivariate Statistical Methods

The PCA is an exploratory tool for the interpretation and evaluation of the water quality [53,54,55,56]. The PCA was based on the eigenvalues of the correlation matrix for the standardized data, while the Varimax rotation was used to maximize the variations expressed by the principal components (PCs). Only PCs with eigenvalues > 1 were retained. The influence of an individual parameter on the hydrogeochemical characteristics was considered strong (>0.70), moderate (0.50–0.70) or weak (0.30–0.50) comparatively to the absolute loading values [6,17]. For the statistical analysis, the concentrations of the chemical parameters below the LODs were considered as half of this value. The seasonal variations of characteristics were emphasized in a heat map obtained after the two-way joining analysis as a clustering method. The heat map is a very suggestive visualization method that shows the results in two dimensions (sample versus parameters), with the different colors depending on the parameters values. Thus, by this approach, it is possible to highlight the seasonal variability of the samples and collection points, respectively. The ANOVA-PCA were applied separately for the water and silty soil samples.

## 5. Conclusions

It was demonstrated that the inductively coupled plasma multielemental spectrometric techniques combined with a multivariate statistical analysis provided an advanced modeling of the water and solid samples in caves compared to the classical geochemical diagrams. The anion contents; different carbon fractions and general characteristics of the water samples (alkalinity, pH, EC and TDS) were also considered in the development of the model. The model was implemented on a case study in Cloșani Cave, Romania. The ANOVA-PCA revealed that the hydrogeochemical characteristics of the Ca^2+^-HCO_3_^−^ water facies were described by five PCs after Varimax rotation: (i) the release of Ca, Mg and several elements of geogenic origin (Ni, Sr and Ba) as a result of calcite-water interactions; (ii) the chemistry of inorganic and organic carbon (TIC and TOC) and the release of elements involved in the redox processes, such as Fe; (iii) the occurrence of Cu, Zn and Sb as trace elements; (iv) the chemistry of inorganic nitrogen (NO_3_^−^ and NH_4_^+^) and occurrence of trace elements (Sn and Cr) involved in the redox processes and (v) the occurrence of K and Mn. Organic carbon fractions (TOC and DOC) have a weak influence on the water characteristics compared to inorganic fraction DIC, in agreement with the Ca^2+^-HCO_3_^−^ water facies. Nevertheless, the microbial films have an important role in the formation of Ba-containing deposits. Although a low concentration of TOC was determined in the water, this was efficient to mobilize Fe and Mn following the reduction process by microbial activity. The occurrence of nitrate in the water is due to natural processes, such as the nitrification of organic matter containing nitrogen, its release by oxidation in the presence of bacteria and leaching in the water. Consequently, the nitrate concentration in the water was influenced by the concentration of organic matter in the water. It was demonstrated that ammonium ion occurs as a result of nitrate microbial reduction processes in the presence of organic matter. The ANOVA-PCA of the water highlighted that the occurrence of Ca and Mg is due to water-carbonate rock interactions in the presence of CO_2_ and less water-sulfate rock interactions. The two-way joining analysis as the clustering method emphasized a seasonal variation, especially in the spring, of the characteristics of Ca^2+^-HCO_3_^−^ water in terms of the pH, Ca, Ba, Sr, HCO_3_^−^, EC, TDS, inorganic carbon (DIC and TIC) and total carbon (DC and TC). The variability of the silty soil was described by four factors after varimax rotation, as follows: (i) REEs together with P and trace elements (Be, Sc, Rb, Y, Zr, Mo, Cs, Th, Ga and Ge); (ii) Ca, Mg and Ba as the major elements and Li, Cr, Mn, Co, Ni, Zn, Cd, Sb, Hf, W, Tl and S as the trace elements; (iii) Na, K and Al as the major elements in aluminosilicates and Sn retained in these rocks and (iv) Sr and V as the trace elements. The ANOVA-PCA clearly highlighted the need to determine the REEs in order to obtain an advanced geochemical characterization of caves. Additionally, ANOVA-PCA, applied on Cloșani Cave, emphasized that the REEs were embedded in mineral deposits together with P and common trace elements (Cr, Mn, Co, Ni, Cu, Zn, As, Cd, Cs, W and Tl), while aluminosilicates do not contain trace elements, except for Sn. The two-way joining analysis emphasized that major elements (Ca, Mg and Al) and trace elements, including several REEs (La, Nd, Th and Ce), showed variability between the silty soil samples. A multi-year study would provide additional information on the hydrogeochemical profiling of caves by unsupervised chemometric tools, and we consider that such a study would be useful to perform.

## Figures and Tables

**Table 1:** Hydrogeochemical characteristics and summary statistics of the water samples seasonally collected from C2.

Param.	Unit	C2wi1 ^1^	C2sp ^1^	C2su^1^	C2au ^1^	C2wi2 ^1^	Min.	Max.	Mean	Median	s ^3^	Skew.	Kurt.
pH		8.1	8.2	7.6	6.6	8.1	6.6	8.2	7.7	8.1	0.7	−1.65	2.44
EC	µS cm^−^^1^	287	144	237	305	168	144	305	228	237	71	−0.19	−2.56
Na	mg L^−^^1^	0.95	0.69	0.87	0.59	0.59	0.59	0.95	0.74	0.69	0.17	0.51	−2.36
Mg	mg L^−^^1^	0.88	0.51	0.75	0.82	0.68	0.51	0.88	0.73	0.75	0.14	−0.91	0.55
K	mg L^−^^1^	0.46	0.47	0.66	0.36	0.34	0.34	0.66	0.46	0.46	0.13	1.11	1.33
Ca	mg L^−^^1^	45.2	26.1	35.2	58.2	41.3	26.1	58.2	41.2	41.3	11.9	0.32	0.46
Al	μg L^−^^1^	11.1	5.1	7.2	0.5	29.2	0.5	29.2	10.6	7.2	11.1	1.59	2.89
Fe	μg L^−^^1^	13.6	8.1	<LOD	13.3	22.9	8.1	22.9	14.5	13.4	6.2	0.95	2.00
Cr	μg L^−^^1^	1.2	<LOD ^2^	1.1	4.6	1.1	1.1	4.6	2.0	1.2	1.7	1.99	3.96
Mn	μg L^−^^1^	1.24	<LOD	2.76	<LOD	<LOD	1.24	2.76	2.00	2.00	1.08	-	-
Ni	μg L^−^^1^	5.2	0.4	3.0	5.4	<LOD	0.4	5.4	3.5	4.1	2.4	−0.94	−0.81
Cu	μg L^−^^1^	2.7	<LOD	1.9	<LOD	<LOD	1.9	2.7	2.3	2.3	0.6	-	-
Zn	μg L^−^^1^	4.0	2.5	4.9	<LOD	2.9	2.5	4.9	3.6	3.5	1.1	0.45	−2.46
Sr	μg L^−^^1^	<LOD	22.0	27.1	29.0	0.3	0.3	29.0	19.6	24.5	13.2	−1.72	2.94
Sn	μg L^−^^1^	0.20	<LOD	0.33	19.5	<LOD	0.20	19.5	6.7	0.30	11.1	1.73	-
Sb	μg L^−^^1^	0.40	<LOD	0.16	<LOD	<LOD	0.16	0.40	0.30	0.30	0.20	-	-
Ba	μg L^−^^1^	12.3	5.8	12.0	11.3	5.1	5.1	12.3	9.3	11.3	3.5	−0.58	−3.14
TOC	mg L^−^^1^	3.0	2.1	2.3	4.8	15.8	2.1	15.8	5.6	3.0	5.8	2.06	4.29
TC	mg L^−^^1^	33.7	13.2	33.2	45.2	17.3	13.2	45.2	28.5	33.2	13.1	−0.02	−1.68
TIC	mg L^−^^1^	30.7	11.1	30.9	40.4	1.6	1.6	40.4	22.9	30.7	16.0	−0.52	−1.74
DOC	mg L^−^^1^	1.4	2.1	1.7	4.8	1.6	1.4	4.8	2.3	1.7	1.4	2.06	4.31
DC	mg L^−^^1^	31.7	12.2	32.7	44.9	20.5	12.2	44.9	28.4	31.7	12.5	−0.03	−0.50
DIC	mg L^−^^1^	30.3	10.1	31.1	40.1	18.9	10.1	40.1	26.1	30.3	11.7	−0.40	−0.79
HCO_3_^−^	mg L^−^^1^	189	85	183	232	134	85	232	165	183	56	−0.48	−0.36
Cl^−^	mg L^−^^1^	0.95	1.35	0.96	0.90	0.79	0.79	1.35	0.99	0.95	0.21	1.65	3.37
NO_3_^−^	mg L^−^^1^	1.59	1.12	1.55	2.92	1.91	1.12	2.92	1.82	1.59	0.68	1.30	2.23
SO_4_^2−^	mg L^−^^1^	10.5	6.3	5.4	8.9	7.5	5.4	10.5	7.7	7.5	2.0	0.41	−1.06
NH_4_^+^	mg L^−^^1^	0.06	0.11	0.12	0.16	0.04	0.04	0.16	0.10	0.11	0.05	0.02	−1.37
TDS	mg L^−^^1^	165	70	75	220	110	70	220	128	110	64	0.77	−1.04

^1^ wi—winter; sp—spring; su—summer; au—autumn; ^2^ LOD—limit of detection (μg L^−1^); Cr—0.22; Mn—0.013; Ni—0.13; Cu—0.21; Zn—0.31; Sr—0.10; Sn—0.06; Sb—0.05; Fe—0.56; ^3^ s—standard deviation of the results.

**Table 2:** Hydrogeochemical characteristics and summary statistics of the water samples seasonally collected from C3.

Param.	Unit	C3wi1 ^1^	C3sp ^1^	C3su ^1^	C3au ^1^	C3wi2 ^1^	Min.	Max.	Mean	Median	s ^3^	Skew.	Kurt.
pH		8.0	7.8	7.3	6.6	7.9	6.6	8.0	7.5	7.8	0.6	−1.28	0.79
EC	µS cm^−^^1^	272	179	172	212	189	172	272	205	189	40	1.55	2.26
Na	mg L^−^^1^	0.82	0.60	0.63	0.67	0.52	0.52	0.82	0.65	0.63	0.11	0.80	1.65
Mg	mg L^−^^1^	0.95	0.48	0.68	0.66	0.74	0.48	0.95	0.70	0.68	0.17	0.34	1.42
K	mg L^−^^1^	0.38	0.13	0.49	0.17	0.23	0.13	0.49	0.28	0.23	0.15	0.69	−1.51
Ca	mg L^−^^1^	50.0	31.2	28.6	35.0	45.4	28.6	50.0	38.0	35.0	9.3	0.49	−2.26
Al	μg L^−^^1^	18.2	4.2	3.3	0.8	5.0	0.8	18.2	6.3	4.2	6.8	1.92	4.03
Fe	μg L^−^^1^	31.6	6.3	76.9	6.7	47.9	6.3	76.9	33.9	31.6	29.8	0.65	−0.73
Cr	μg L^−^^1^	0.9	<LOD ^2^	1	2.2	1.3	0.9	2.2	1.4	1.2	0.6	1.55	2.23
Mn	μg L^−^^1^	1.48	<LOD	1.37	0.04	<LOD	0.04	1.48	0.96	1.37	0.80	−1.70	-
Ni	μg L^−^^1^	4.1	1.2	2.7	2.9	<LOD	1.2	4.1	2.7	2.8	1.2	−0.46	1.50
Cu	μg L^−^^1^	0.8	<LOD	1.1	<LOD	<LOD	0.8	1.1	1.0	1.0	0.2	-	-
Zn	μg L^−^^1^	2.5	1.4	6.0	3.7	5.0	1.4	6.0	3.7	3.7	1.9	−0.02	−1.52
Sr	μg L^−^^1^	<LOD	23.9	23.2	17.0	34.3	17.0	34.3	24.6	23.6	7.2	0.85	1.87
Sn	μg L^−^^1^	<LOD	0.2	<LOD	1.3	<LOD	0.2	1.3	0.8	0.8	0.8	-	-
Sb	μg L^−^^1^	<LOD	<LOD	<LOD	<LOD	<LOD	-	-	-	-	-	-	-
Ba	μg L^−^^1^	11.3	6.1	6.3	5.6	5.0	5.0	11.3	6.9	6.1	2.5	2.02	4.30
TOC	mg L^−^^1^	2.2	3.1	1.2	3.0	16.6	1.2	16.6	5.2	3.0	6.4	2.16	4.73
TC	mg L^−^^1^	30.8	20.0	25.9	34.2	19.2	19.2	34.2	26.0	25.9	6.6	0.18	−2.31
TIC	mg L^−^^1^	28.6	16.9	24.7	31.2	2.6	2.6	31.2	20.8	24.7	11.5	−1.21	0.93
DOC	mg L^−^^1^	1.8	2.8	1.1	2.5	2.5	1.1	2.8	2.1	2.5	0.7	−1.01	−0.10
DC	mg L^−^^1^	30.1	18.7	22.2	33.67	22.4	18.7	33.7	25.4	22.4	6.2	0.52	−1.85
DIC	mg L^−^^1^	28.3	15.9	21.11	31.18	19.9	15.9	31.2	23.3	21.1	6.3	0.28	−1.89
HCO_3_^−^	mg L^−^^1^	177	110	128	164	134	110	177	143	134	27	0.23	−1.75
Cl^−^	mg L^−^^1^	0.98	1.28	0.59	0.91	0.82	0.59	1.28	0.92	0.91	0.25	0.34	1.11
NO_3_^−^	mg L^−^^1^	0.95	0.64	0.69	1.41	1.39	0.64	1.41	1.02	0.95	0.37	0.21	−2.99
SO_4_^2-^	mg L^−^^1^	10.3	4.0	3.6	5.1	5.1	3.6	10.3	5.6	5.1	2.7	1.92	3.91
NH_4_^+^	mg L^−^^1^	0.04	0.04	0.04	0.14	0.06	0.04	0.14	0.06	0.04	0.04	1.79	3.20
TDS	mg L^−^^1^	165	92	58	190	100	58	190	121	100	55	0.34	−1.98

^1^ wi—winter; sp—spring; su—summer; au—autumn; ^2^ LOD—limit of detection (μg L^−1^); Cr—0.22; Mn—0.013; Ni—0.13; Cu—0.21; Zn—0.31; Sr—0.10; Sn—0.06; Sb—0.05; Fe—0.56; ^3^ s—standard deviation of the results.

**Table 3:** Determination coefficients between the concentrations (mEq L^−1^) of the major ions in the water samples collected from Cloșani Cave.

Cation	Anion					
	Cl^−^		HCO_3_^−^		SO_4_^2−^	
	All Samples	without Outliers ^1^	All Samples	without Outliers ^2^	All Samples	without Outliers ^3^
Ca^2+^	0.0783	0.0009	0.6250	0.2338	0.5248	0.5720
Mg^2+^	0.2227	0.4234	0.6148	0.6183	0.5577	0.7205
Na^+^	0.0216	0.6777	0.1359	0.7905	0.2718	0.2312
K^+^	0.0217	0.2605	0.0421	0.3079	0.0405	0.0008

^1^ Without samples collected in the spring (C2 and C3 sampling points) and summer (C3 sampling point); ^2^ without samples collected in the spring and autumn (C2 sampling point); ^3^ without samples collected in the spring (C2 and C3 sampling points).

**Table 4:** Concentrations of the elements (mg kg^−^^1^) in the silty soil samples collected from the C1 sampling point and the summary statistics.

Param.	C11	C12	C13	C14	C15	Min.	Max.	Mean	Median	s ^1^	Skew.	Kurt.
Na	211	477	540	124	111	111	540	293	211	202	0.51	−2.86
Mg	6547	8290	7810	9707	5873	5873	9707	7645	7810	1503	0.26	−0.77
K	2073	2973	3420	2333	3433	2073	3433	2847	2973	623	−0.35	−2.56
Ca	9567	17,443	13,463	16,687	10,023	9567	17,443	13,437	13,463	3648	0.01	−2.85
Al	29,020	39,700	40,600	26,100	27,557	26,100	40,600	32,595	29,020	6981	0.52	−3.13
Fe	35,067	40,167	43,433	44,340	39,667	35,067	44,340	40,535	40,167	3663	−0.72	0.16
P	2092	2690	1326	1178	9990	1178	9990	3455	2092	3704	2.09	4.46
S	72.0	73.8	42.6	35.9	30.9	30.9	73.8	51.0	42.6	20.4	0.43	−3.06
Li	14.9	10.2	23.2	10.2	27.8	10.2	27.8	17.3	14.9	7.9	0.55	−2.12
Be	0.61	0.36	0.51	0.47	1.33	0.36	1.33	0.66	0.51	0.39	1.95	4.00
Sc	3.57	2.71	3.63	2.92	10.17	2.71	10.17	4.60	3.57	3.14	2.15	4.68
V	24.4	26.2	46.5	36.0	47.1	24.4	47.1	36.0	36.0	10.8	−0.01	−2.93
Cr	13.8	16.9	28.2	26.4	25.4	13.8	28.2	22.1	25.4	6.4	−0.63	−2.41
Mn	388	317	580	512	598	317	598	479	512	123	−0.51	−2.09
Co	5.6	4.2	8.6	12.4	8.6	4.2	12.4	7.9	8.6	3.1	0.41	−0.26
Ni	11.1	8.3	19.0	21.5	17.0	8.3	21.5	15.4	17.0	5.5	−0.38	−2.01
Cu	11.7	7.9	17.6	11.0	17.3	7.9	17.6	13.1	11.7	4.2	0.08	−2.22
Zn	30.0	37.4	41.0	36.0	46.7	30.0	46.7	38.2	37.4	6.2	0.10	0.40
As	8.58	6.53	4.08	2.10	3.84	2.10	8.58	5.02	4.08	2.54	0.53	−0.75
Rb	14.8	11.0	17.7	11.0	35.0	11.0	35.0	17.9	14.8	9.9	1.82	3.44
Sr	40.6	26.2	56.7	18.3	42.4	18.3	56.7	36.8	40.6	14.9	0.06	−0.83
Y	8.90	5.00	6.23	7.27	25.57	5.00	25.57	10.59	7.27	8.49	2.08	4.44
Zr	2.24	0.68	1.19	1.10	2.71	0.68	2.71	1.58	1.19	0.85	0.53	−1.98
Mo	0.18	0.02	0.15	0.17	0.37	0.02	0.37	0.18	0.17	0.12	0.60	2.04
Cd	0.17	0.04	0.20	0.13	0.11	0.04	0.20	0.13	0.13	0.06	−0.63	0.22
Pb	8.12	8.17	13.36	11.73	7.27	7.27	13.36	9.73	8.17	2.66	0.73	−2.02
Sn	0.51	1.87	0.44	0.28	0.80	0.28	1.87	0.78	0.51	0.64	1.79	3.30
Sb	0.12	0.01	0.03	0.03	0.11	0.01	0.12	0.06	0.03	0.05	0.53	−3.03
Ba	161.2	66.4	78.8	117.2	165.1	66.4	165.1	117.7	117.2	45.5	−0.03	−2.80
Cs	0.9	0.9	0.8	0.5	1.5	0.5	1.5	0.9	0.9	0.3	1.08	2.51
La	15.2	13.2	13.8	15.0	38.6	13.2	38.6	19.2	15.0	10.9	2.21	4.89
Ce	33.2	33.7	36.1	37.4	82.8	33.2	82.8	44.6	36.1	21.4	2.20	4.87
Pr	3.9	3.6	3.6	4.0	9.3	3.6	9.3	4.9	3.9	2.5	2.21	4.92
Nd	14.6	13.7	13.5	13.5	31.6	13.5	31.6	17.4	13.7	8.0	2.22	4.93
Sm	2.9	3.1	2.5	2.6	6.0	2.5	6.0	3.4	2.9	1.5	2.09	4.47
Eu	0.5	0.6	0.5	0.5	1.1	0.5	1.1	0.7	0.5	0.3	2.20	4.87
Gd	3.4	2.7	2.5	2.7	6.6	2.5	6.6	3.6	2.7	1.7	2.01	4.10
Tb	0.39	0.35	0.34	0.35	0.84	0.34	0.84	0.45	0.35	0.22	2.19	4.81
Dy	1.87	1.73	1.61	1.77	4.30	1.61	4.30	2.26	1.77	1.15	2.20	4.88
Ho	0.33	0.30	0.28	0.32	0.79	0.28	0.79	0.40	0.32	0.22	2.19	4.84
Er	1.00	0.80	0.75	0.82	2.08	0.75	2.08	1.09	0.82	0.56	2.08	4.38
Yb	0.88	0.77	0.73	0.77	1.78	0.73	1.78	0.99	0.77	0.45	2.15	4.69
Lu	0.14	0.11	0.10	0.11	0.25	0.10	0.25	0.14	0.11	0.06	1.90	3.58
Hf	0.08	0.02	0.03	0.03	0.06	0.02	0.08	0.05	0.03	0.02	1.12	0.13
W	0.18	0.03	0.03	0.03	0.05	0.03	0.18	0.06	0.03	0.07	2.14	4.64
Tl	0.22	0.14	0.11	0.09	0.18	0.09	0.22	0.15	0.14	0.05	0.55	−0.89
Th	27.33	14.50	16.40	22.57	32.67	14.50	32.67	22.69	22.57	7.54	0.28	−1.66
Ga	4.07	3.79	4.87	3.37	9.93	3.37	9.93	5.21	4.07	2.70	2.02	4.17

^1^ s—standard deviation of the results.

**Table 5:** Powder X-ray diffraction results of the silty soil samples (<150 μm).

Mineral Group	Mineral Species	Chemical Formula	Abundance ^1^				
			C11	C12	C13	C14	C15
Oxide	Quartz	SiO_2_	++	++	++	++	++
Carbonates	Calcite	CaCO_3_	+	+	+	+	+
Silicates	Albite	NaAlSi_3_O_8_	+	+	++	++	±
	Muscovite calcian	(K,Ca,Na)(Al,Mg,Fe)_2_(Si,Al)_4_O_10_(OH)_2_	+	+	+	+	+
	Illite	(K,H_30_)Al_2_(Si_3_,Al)O_10_(OH)_2_ × H_2_O	±	±	±	±	±
	Microcline-(Potassium-feldspar)	KAlSi_3_O_8_	±	±	±	±	±
	Gismondine	CaAl_2_Si_2_O_8_ × 4(H_2_O)	±	±	±	±	±
Sulfate	Gypsum	CaSO_4_ × 2H_2_O	±	±	±	±	±

^1^ + + Major species (20–50%); + Minor species (5–20%); ± Trace species (<5%).

**Table 6:** Factor loadings after Varimax rotation, and eigenvalues describing the variances of the chemical compositions in the water collected from Cloșani Cave ^1^.

Parameters	Factor 1	Factor 2	Factor 3	Factor 4	Factor 5
pH	−0.61	−0.40	0.27	−0.46	0.07
EC	0.61	0.34	0.28	0.27	0.22
Na	0.22	0.49	0.23	0.03	0.46
Mg	**0.80**	0.15	0.33	−0.05	0.28
K	0.19	−0.23	0.30	0.07	**0.79**
Ca	**0.88**	−0.25	−0.03	0.30	−0.19
Al	0.05	**−0.89**	−0.11	−0.06	0.14
Fe	0.09	**−0.96**	−0.08	−0.08	0.03
Cr	*0.56*	0.49	−0.08	*0.60*	−0.08
Mn	0.00	0.26	0.11	−0.16	**0.91**
Ni	**0.77**	−0.46	−0.03	0.37	−0.22
Cu	0.01	0.23	**0.82**	−0.09	0.50
Zn	0.20	*−0.58*	**0.72**	−0.15	−0.19
Sr	**0.83**	−0.10	0.03	0.28	−0.00
Sn	0.48	0.11	−0.19	**0.72**	−0.12
Sb	0.02	0.15	**0.92**	−0.04	0.17
Ba	**0.77**	−0.30	0.10	0.24	0.33
TOC	0.19	**−0.91**	0.07	−0.03	−0.25
TC	**0.71**	0.48	0.11	0.39	0.26
TIC	0.45	**0.75**	0.05	0.30	0.31
DOC	0.27	0.10	−0.26	**0.76**	−0.40
DC	**0.77**	0.30	0.10	0.44	0.25
DIC	**0.78**	0.31	0.14	0.38	0.30
HCO_3_^−^	**0.92**	−0.14	−0.08	0.25	−0.14
Cl^−^	**−0.77**	0.24	−0.03	0.34	−0.21
NO_3_^−^	*0.50*	−0.32	0.12	**0.75**	0.09
SO_4_^2−^	0.29	−0.06	0.29	0.10	0.08
NH_4_^+^	0.14	0.20	0.02	**0.89**	0.09
TDS	*0.66*	0.18	0.09	0.40	−0.24
Eigenvalue	12.6	5.8	4.8	1.9	1.4
Total var. (%)	43.4	20.1	16.7	6.7	4.7
Cumulative (%)	43.4	63.5	80.2	86.9	91.6

^1^ Strong relationship loading values > 0.70 are in bold face, moderate values between 0.50 and 0.70 are marked in italics and the values below <0.50 corresponding to a weak relationship are written with regular font [6,17].

**Table 7:** Factor loadings after the Varimax rotation, and eigenvalues describing the variances of the elemental patterns of the silty soil samples collected from Cloșani Cave ^1^.

Element	Factor 1	Factor 2	Factor 3	Factor 4
Na	−0.37	−0.13	**−0.84**	0.37
Mg	−0.19	**0.96**	0.21	0.01
K	−0.41	0.01	**−0.91**	−0.01
Ca	−0.24	**0.81**	−0.47	0.26
Al	−0.28	−0.28	**−0.91**	0.15
Fe	−0.18	**0.91**	0.36	−0.04
P	**1.00**	0.02	−0.05	−0.00
Li	0.09	**0.87**	−0.39	0.29
Be	**0.96**	0.05	0.26	0.06
Sc	**0.98**	−0.04	0.16	0.10
V	0.49	*−0.66*	0.35	0.44
Cr	0.12	**0.98**	−0.13	0.09
Mn	−0.19	**0.88**	−0.30	−0.31
Co	0.18	**0.98**	0.02	−0.07
Ni	0.11	**0.99**	−0.00	0.03
Cu	0.10	**0.99**	−0.04	−0.04
Zn	0.06	**0.97**	0.05	−0.23
As	−0.09	**0.88**	−0.46	0.07
Rb	**0.94**	−0.04	0.19	0.26
Sr	0.09	0.16	−0.35	**0.92**
Y	**0.98**	0.02	0.21	−0.02
Zr	**0.74**	0.46	0.49	0.06
Mo	**0.94**	−0.01	0.35	−0.03
Cd	0.01	**0.96**	0.26	−0.09
Pb	*−0.69*	0.44	0.43	−0.39
Sn	0.08	0.09	**−0.99**	−0.11
Sb	*0.58*	**0.71**	0.38	0.15
Ba	−0.33	*0.56*	**−0.72**	0.23
Cs	**0.93**	0.21	−0.15	0.27
W	−0.04	**0.96**	0.27	0.08
Tl	0.38	**0.92**	0.06	0.07
Th	**0.73**	0.33	0.54	−0.25
Ga	**0.97**	−0.07	0.10	0.21
S	−0.46	**0.72**	−0.52	−0.01
La	**0.99**	−0.06	0.14	−0.02
Ce	**0.98**	−0.15	0.12	−0.00
Pr	**0.99**	−0.08	0.12	−0.04
Nd	**1.00**	−0.02	0.09	0.01
Sm	**1.00**	0.02	−0.05	−0.02
Eu	**1.00**	−0.05	0.01	0.01
Gd	**0.99**	0.12	0.12	−0.04
Tb	**0.99**	0.01	0.11	−0.02
Dy	**0.99**	−0.02	0.09	−0.04
Ho	**0.99**	−0.02	0.10	−0.06
Er	**0.99**	0.08	0.13	−0.04
Yb	**0.99**	0.04	0.11	−0.02
Lu	**0.98**	0.18	0.12	−0.02
Hf	0.34	**0.85**	0.30	0.26
Eigenvalue	25.6	15.6	6.0	1.8
Total Variance (%)	52.2	31.9	12.3	3.6
Cumulative (%)	52.2	84.1	96.4	100

^1^ Strong relationship loading values >0.70 are in bold face, moderate values between 0.50 and 0.70 are marked in italics and the values below <0.50 corresponding to a weak relationship are written with regular font [6,17].

## Data Availability

The data presented in this study is available upon request from the corresponding author.

## Supplementary Materials

The followings are available online. Table S1. Operating conditions for the OPTIMA 5300 DV ICP-OES Perkin Elmer spectrometer for Al, Na, K, Ca, Mg, P, S and Fe determination in the water and silty soil samples. Table S2. Operating conditions for the ELAN DRCII Perkin Elmer ICP-MS spectrometer for multielemental determination in the water and silty soil samples. Table S3. Limits of detection for multielemental determination by ICP-OES. Table S4. Limits of detection for multielemental determination by ICP-MS. Tables S5–S8. Validation of the ICP-OES and ICP-MS methods for multielemental determination in the water, soil and sediments. Table S9. Operating conditions for the 761 Compact IC Metrohm ion chromatograph. Table S10. Limits of detection for anion determination in the water by ion chromatography. Figure S1. Relationship between the major ions in the water samples collected from Cloșani Cave considering all the samples: (a) Ca^2+^-Cl^−^, (b) Ca^2+^-HCO_3_^−^, (c) Ca^2+^-SO_4_^2−^, (d) Mg^2+^-Cl^−^, (e) Mg^2+^-HCO_3_^−^, (f) Mg^2+^-SO_4_^2−^, (g) Na^+^-Cl^−^, (h) Na^+^-HCO_3_^−^, (i) Na^+^-SO_4_^2−^, (j) K^+^-Cl^−^, (k) K^+^-HCO_3_^−^ and (l) K^+^-SO_4_^2−^. C2 and C3—sampling points. Figure S2. Relationship between the major ions in the water samples collected from Cloșani Cave after the elimination of the outliers caused by the seasonal variability: (a) Ca^2+^-Cl^−^, (b) Ca^2+^-HCO_3_^−^, (c) Ca^2+^-SO_4_^2−^, (d) Mg^2+^-Cl^−^, (e) Mg^2+^-HCO_3_^−^, (f) Mg^2+^-SO_4_^2−^, (g) Na^+^-Cl^−^, (h) Na^+^-HCO_3_^−^, (i) Na^+^-SO_4_^2−^, (j) K^+^-Cl^−^, (k) K^+^-HCO_3_^−^ and (l) K^+^-SO_4_^2−^. C2 and C3—sampling points.

## Sample Availability

CRM samples are available from the manufacturer, while cave samples are available from the authors.
