# Supplementary material for: Application of Inductively Coupled Plasma Spectrometric Techniques and Multivariate Statistical Analysis in the Hydrogeochemical Profiling of Caves—Case Study Cloșani, Romania
Source: Molecules. 2021 Nov 10;26(22):6788. doi: 10.3390/molecules26226788 (PMC8619360; doi:10.3390/molecules26226788)
Supplement: Supplementary file 1 [file molecules-26-06788-s001.zip › molecules-1420078-supplementary.pdf]

Supplementary Material

Application of Inductively Coupled Plasma Spectrometric Techniques and Multivariate Statistical Analysis in the Hydro-geochemical Profiling of Caves–Case Study Cloșani, Romania

Anamaria Iulia Torok [1], Erika Andrea Levei [1], Silviu Constantin [2,3,4], Oana Teodora Moldovan [3,4,5], Marin Senila [1], Oana Cadar [1], Dorina Casoni [6,7], Simion Bogdan Angyus [1,6,7], Claudiu Tanaselia [1], Eniko Covaci [6,7] and Tiberiu Frentiu [6,7]*

1 INCDO–INOE 2000 National Institute for Research and Development of Optoelectronics,, Research Institute for Analytical Instrumentation subsidiary, Donath 67, 400293 Cluj-Napoca, Romania; iulia.torok@icia.ro (A.I.T.); erika.levei@icia.ro (E.A.L.); marin.senila@icia.ro (M.S.); oana.cadar@icia.ro, (O.C.); bogdan.angyus@gmail.com (S.B.A.); claudiu.tanaselia@icia.ro (C.T.);

2 Department of Geospeleology and Paleontology, Emil Racovita Institute of Speleology, Calea 13 Septembrie, 050711 Bucharest, Romania; silviu.constantin@iser.ro

3 Romanian Institute of Science and Technology, Saturn 24-26, 400504 Cluj-Napoca, Romania

4 Centro Nacional de Investigación sobre la Evolución Humana, Paseo Sierra de Atapuerca 3, 09002 Burgos, Spain

5 Department of Cluj-Napoca, Emil Racovita Institute of Speleology, Cluj Department, Clinicilor 5, 400006 Cluj-Napoca, Romania; oanamol35@gmail.com

6 Department of Chemistry, Faculty of Chemistry and Chemical Engineering, Babes-Bolyai University, Arany Janos 11, 400028 Cluj-Napoca, Romania; dorina.casoni@ubbcluj.ro (D.C.); eniko.covaci@ubbcluj.ro (E.C.); tiberiu.frentiu@ubbcluj.ro (T.F.);

7 Research Center for Advanced Chemical Analysis, Instrumentation and Chemometrics–Analytica, Babes-Bolyai University, Arany Janos 11, 400028 Cluj-Napoca, Romania

* Correspondence: tiberiu.frentiu@ubbcluj.ro (T.F.); Tel.: +40-264-593833

This online resource contains the following data:

1. Operating conditions for OPTIMA 5300 DV ICP–OES Perkin Elmer spectrometer for Al, Na, K, Ca, Mg, P, S and Fe determination in water and silty soil samples (Table S1)
2. Operating conditions for ELAN DRCII Perkin Elmer ICP–MS spectrometer for multielemental determination in water and silty soil samples (Table S2)
3. Limits of detection for multielemental determination by ICP–OES (Table S3)
4. Limits of detection for multielemental determination by ICP–MS (Table S4)
5. Validation of ICP-OES and ICP-MS methods for multielemental determination in water, soil and sediments (Tables S5–S8)
6. Operating conditions for 761 Compact IC Metrohm ion chromatograph (Table S9);
7. Limits of detection for anions determination in water by ion chromatography (Table S10)
8. Relationship between major ions in water samples collected from Cloșani Cave considering all samples (a) Ca^{2+} – Cl^{-}; (b) Ca^{2+} – HCO_3^{-}; (c) Ca^{2+} – SO_4^{2-}; (d) Mg^{2+} – Cl^{-}; (e) Mg^{2+} – HCO_3^{-}; (f) Mg^{2+} – SO_4^{2-}; (g) Na^{+} – Cl^{-}; (h) Na^{+} – HCO_3^{-} (i) Na^{+} – SO_4^{2-}; (j) K^{+} – Cl^{-}; (k) K^{+} – HCO_3^{-}; (l) K^{+} – SO_4^{2-}. C2 and C3 – sampling points.
9. Relationship between major ions in water samples collected from Cloșani Cave after the elimination of outliers caused by the seasonal variability (a) Ca^{2+} – Cl^{-}; (b) Ca^{2+} – HCO_3^{-}; (c) Ca^{2+} – SO_4^{2-}; (d) Mg^{2+} – Cl^{-}; (e) Mg^{2+} – HCO_3^{-}; (f) Mg^{2+} – SO_4^{2-}; (g) Na^{+} – Cl^{-}; (h) Na^{+} – HCO_3^{-}; (i) Na^{+} – SO_4^{2-}; (j) K^{+} – Cl^{-}; (k) K^{+} – HCO_3^{-}; (l) K^{+} – SO_4^{2-}. C2 and C3 – sampling points.

1. Operating conditions for ICP–OES

Table S1. Operating conditions for OPTIMA 5300 DV ICP-OES Perkin Elmer spectrometer for Al, Na, K, Ca, Mg, P, S and Fe determination in water and silty soil samples

Parameter	Settings
Plasma power (W)	1300
Ar flow rate (L min^{-1})	
- Plasma support	15
- Auxiliary gas	2.0
- Nebulizer gas	0.8
Plasma viewing	Axial viewing for Al, Na, K, Ca, Mg, P and S and radial viewing for Fe.
Sample introduction (mL min^{-1})	1.5
Calibration range (mg L^{-1})	0–20 (n = 7 points linear calibration curve)
Wavelength (nm)	Al 396.152; Na 589.592; K 766.490; Mg 285.213; Ca 317.933; P 213.618; S 180.731; Fe 238.204.

2. Operating conditions for ICP-MS

Table S2. Operating conditions for ELAN DRCII Perkin Elmer ICP–MS spectrometer for multielemental determination in water and silty soil samples

Parameter	Settings
Plasma power (W)	1400
Ar flow rate (L min^{-1})	
- Plasma support	15
- Auxiliary gas	1.2
- Nebulizer gas	0.96
Analog stage voltage (V)	–1850
Pulse stage voltage (V)	1050
Scan Mode	Peak Hopping
Dwell Time per Amu (ms)	200
Integration Time (ms)	4000
Detector	Analog
Isotopes and DRC operation	Without DRC (Rpq = 0.25, reaction gas absent): ^{7}Li, ^{9}Be, ^{45}Sc, ^{51}V, ^{52}Cr, ^{55}Mn, ^{59}Co, ^{60}Ni, ^{63}Cu, ^{66}Zn, ^{85}Rb, ^{88}Sr, ^{89}Y, ^{90}Zr, ^{111}Cd, ^{208}Pb, ^{118}Sn, ^{121}Sb, ^{133}Cs, ^{184}W, ^{205}Tl, ^{232}Th, ^{69}Ga, ^{139}La, ^{140}Ce, ^{141}Pr, ^{153}Eu, ^{159}Tb, ^{165}Ho, ^{166}Er, ^{175}Lu, ^{178}Hf<br>Without DRC (Rpq = 0.25, reaction gas absent) default mathematical modelling isobaric interference: ^{98}Mo (- 0.109613 × ^{101}Ru); ^{138}Ba (- 0.000901 ×^{139}La); ^{142}Nd (- 0.125653 × ^{140}Ce); ^{152}Sm (- 0.012780 × ^{157}Gd); ^{158}Gd (- 0.004016 × ^{163}Dy); ^{164}Dy (- 0.047902 × ^{166}Er); ^{174}Yb (- 0.005865 × ^{178}Hf)<br>With DRC (RPq = 0.45; O_2 reaction gas flow rate 0.4 mL min^{-1}): $(^{75}As^{16}O)^{+}$
Isotopic internal standard	No

3. Limits of detection for multielemental determination by ICP-OES

Table S3. Limits of detection for multielemental determination by ICP–OES

Element	Wavelength (nm)	Plasma viewing	LOD	
			Water (μg L^{-1})	Silty soil (mg kg^{-1}) [1]
Na	589.592	Axial	8	0.27
K	766.490	Axial	3	0.10
Mg	285.213	Axial	9	0.30
Ca	317.933	Axial	4	0.13
Al	396.152	Axial	2	0.07
P	213.618	Axial	50	1.67
S	180.731	Axial	50	1.67
Fe	238.204	Radial	8	0.27

[1] Limits of detection in solid calculated for 3 g digested sample made up to 100 mL.

4. Limits of detection for multielemental determination by ICP–MS

Table S4. Limits of detection for multielemental determination by ICP–MS

Element	Isotope	LOD	
		Water (µg L^{-1})	Silty soil (mg kg^{-1}) [1]
Li	^{7}Li	0.16	0.005
Be	^{9}Be	0.28	0.009
Sc	^{45}Sc	0.13	0.004
V	^{51}V	0.16	0.005
Cr	^{52}Cr	0.22	0.007
Mn	^{55}Mn	0.013	0.001
Co	^{59}Co	0.15	0.005
Ni	^{60}Ni	0.13	0.005
Cu	^{63}Cu	0.21	0.007
Zn	^{66}Zn	0.31	0.010
As [2]	$^{75}As^{16}O$	0.27	0.009
Rb	^{85}Rb	0.21	0.007
Sr	^{88}Sr	0.10	0.004
Y	^{89}Y	0.03	0.001
Zr	^{90}Zr	0.06	0.002
Mo	^{98}Mo	0.15	0.005
Cd	^{111}Cd	0.07	0.002
Pb	^{208}Pb	0.11	0.004
Sn	^{118}Sn	0.06	0.002
Sb	^{121}Sb	0.05	0.002
Ba	^{138}Ba	0.16	0.005
Cs	^{133}Cs	0.61	0.020
W	^{184}W	0.05	0.0017
Tl	^{205}Tl	0.49	0.016
Th	^{232}Th	0.57	0.019
Ga	^{69}Ga	0.090	0.003
La	^{139}La	0.025	0.0009
Ce	^{140}Ce	0.030	0.0010
Pr	^{141}Pr	0.026	0.0009
Nd	^{142}Nd	0.050	0.0016
Sm	^{152}Sm	0.051	0.0017
Eu	^{153}Eu	0.031	0.0010
Gd	^{158}Gd	0.053	0.0018
Tb	^{159}Tb	0.025	0.0008
Dy	^{164}Dy	0.041	0.0014
Ho	^{165}Ho	0.023	0.0008
Er	^{166}Er	0.032	0.0011
Yb	^{174}Yb	0.030	0.0010
Lu	^{175}Lu	0.022	0.0008
Hf	^{178}Hf	0.022	0.0008

[1] Limits of detection in solid calculated for 3 g digested sample made up to 100 mL.

[2] Arsenic determined by DRC–ICP–MS at $^{75}As^{16}O^{+}$ polyatomic ion (RPq = 0.45, O_2 reaction gas 0.4 mL min^{-1})

5. Validation of ICP–OES and ICP–MS methods for multielemental determination in water, soil and sediments

Table S5. Results (μg L^{-1}, n = 3) for the analysis of certified reference material SRM 1643f Trace Elements in Water by ICP–OES and ICP–MS methods

Element	Certified Value ± U [1]	Found Value ± C.I. [2]	Recovery ± C.I. (%)
		**ICP-OES**	
Na	18830 ± 250	18557 ± 725	99 ± 4
K	1932.6 ± 9.4	1854.5 ± 83.5	96 ± 5
Ca	29430 ± 330	2822`0 ± 1360	96 ± 5
Mg	7454 ± 60	7288 ± 210	98 ± 3
Al	133.8 ± 1.2	135.0 ± 3.7	101 ± 3
Fe	93.44 ± 0.78	97.60 ± 2.93	104 ± 3
		**ICP-MS**	
Cr	18.50 ± 0.10	19.50 ± 0.80	105 ± 4
Mn	37.14 ± 0.60	37.10 ± 1.50	100 ± 4
Ni	59.8 ± 1.4	52.8 ± 2.5	88 ± 5
Cu	21.66 ± 0.71	21.70 ± 0.80	100 ± 4
Zn	74.4 ± 1.7	72.4 ± 2.6	97 ± 4
Sr	314 ± 19	344 ± 14	110 ± 4
Ba	518.2 ± 7.3	588.0 ± 26.6	113 ± 5
Sb	55.45 ± 0.40	58.25 ± 0.75	105 ± 3

[1] U is the expanded uncertainty for 95% confidence level (k = 2). [2] C.I. is the confidence interval for 95% confidence level (n = 3).

Table S6. Results (mg kg^{-1}, n = 3) for the analysis of soil and sediment CRMs by ICP–OES

Element	CRM 048-50G Trace Metals – Sand 1		Metranal 32 Light Sandy Soil		NCS ZC 73006 Soil		GBW 07404 Soil		LRAC6625 Loamy Clay		Recovery ± C.I. (%)
	Certified Value ± U [1]	Found Value ± C.I. [2]	Certified Value ± U [1,3]	Found Value ± C.I. [2]	Certified Value ± U [1]	Found Value ± C.I. [2]	Certified Value ± U [1]	Found Value ± C.I. [2]	Certified Value ± U [1]	Found Value ± C.I.[2]	
Na	8290 ± 266	8140 ± 516	(10760)	9650 ± 1190	9350 ± 370	8900 ± 740	816 ± 148	742 ± 148	1417 ± 28	1502 ± 101	96 ± 12
K	11400 ± 308	11900 ± 988	(38580)	36350 ± 2970	17510 ± 300	18920 ± 1190	7640 ± 445	7270 ± 668	6087 ± 141	6134 ± 332	101 ± 8
Ca	6410 ± 202	6630 ± 330	(8900)	9200 ± 1110	11350 ± 300	11000 ± 1040	1930 ± 300	1780 ± 370	4272 ± 66	4218 ± 84	99 ± 12
Mg	7030 ± 288	6910 ± 620	(14100)	12910 ± 1190	13350 ± 450	12830 ± 820	3635 ± 370	3264 ± 445	1827 ± 43	1979 ± 112	95 ± 9
Fe	12300 ± 304	11700 ± 711	(27970)	26260 ± 2820	47780 ± 520	46370 ± 2450	76400 ± 820	74480 ± 5790	12920 ± 290	12550 ± 410	96 ± 7
Al	12900 ± 1060	12500 ± 1120	(104000)	100600 ± 10460	113500 ± 740	116470 ± 8160	173970 ± 1410	148300 ± 16540	11450 ± 620	760 ± 100	96 ± 9
P	288 ± 14.7	275 ± 16.8	(2357)	2240 ± 105	560 ± 18	553 ± 31	695 ± 28	724 ± 38	294 ± 26	304 ± 34	99 ± 7
S	-	-	-	-	176 ± 22	169 ± 18	180 ± 36	190 ± 23	-	-	101 ± 11

[1] U is the expanded uncertainty for 95% confidence level (k = 2). [2] C.I. is the confidence interval for 95% confidence level (n = 3). [3] Indicative values

Table S7. Results (mg kg^{-1}, n = 3) for the analysis of soil and sediment CRMs by ICP–MS

Element	CRM 048-50G Trace Metals – Sand 1		Metranal 32 Light Sandy Soil		BCR-280R Lake Sediment		NCS DC 78301 River Sediment		Recovery ± C.I. (%)
	Certified Value ± U [1]	Found Value ± C.I. [2]	Certified Value ± U [1]	Found Value ± C.I. [2]	Certified Value ± U [1]	Found Value ± C.I. [2]	Certified Value ± U [1]	Found Value ± C.I. [2]	
V	101 ± 2.54	100 ± 4.19	44.6 ± 3.4	41.3 ± 5.9	-		(96) [3]	98 ± 13	98 ± 12
Cr	239 ± 4.79	231 ± 15.79	147 ± 8	162 ± 10	126 ± 7	119 ± 12	90 ± 8	56 ± 10	91 ± 11
Mn	1160 ± 37.9	1083 ± 22.1	531 ± 19	592 ± 34	-		975 ± 34	979 ± 149	102 ± 9
Co	177 ± 4.56	172 ± 10.69	11.1 ± 0.5	11.5 ± 1.2	16.8 ± 0.9	17.0 ± 1.8	16.5 ± 1.5	15.9 ± 2.2	100 ± 11
Ni	100.00 ± 2.65	95.30 ± 5.72	40.1 ± 1.2	41.3 ± 4.8	69 ± 5	70 ± 7	(32) [3]	32 ± 4	100 ± 10
Cu	277 ± 6.03	270 ± 5.83	27.3 ± 0.7	30.1 ± 1.7	53 ± 6	54 ± 6	53 ± 6	52 ± 7	102 ± 9
Zn	724 ± 21.2	713.3 ± 11.2	64 ± 1.5	68.3 ± 5.9	224 ± 25	220 ± 23	(251) [3]	246 ± 32	100 ± 9
As	123 ± 3.40	121 ± 6.93	26.1 ± 1.1	29.5 ± 2.8	33.4 ± 2.9	32.1 ± 5.1	56 ± 10	55 ± 9	101 ± 13
Sb	139 ± 13.9	145 ± 23.8	-		-		-		104 ± 16
Sr	213 ± 9.77	207 ± 13.16	-		-		-		97 ± 6
Cd	140 ± 3.28	136 ± 7.87	0.28 ± 0.03	0.25 ± 0.05	0.85 ± 0.10	0.81 ± 0.11	2.45 ± 0.3	2.25 ± 0.3	93 ± 14
Pb	86.9 ± 2.42	82.8 ± 5.40	35.5 ± 0.9	37.1 ± 2.5	-		79 ± 12	78 ± 11	100 ± 10
Ba	235 ± 6.30	222 ± 13.00	(99.1) [3]	105.0 ± 7.9	-		375 ± 22	384 ± 36	101 ± 8
Li	92.1 ± 12.2	86.5 ± 6.1	-		-				94 ± 7
Mo	138 ± 3.29	127 ± 6.51	-		-				92 ± 5

[1] U is the expanded uncertainty for 95% confidence level (k = 2). [2] C.I. is the confidence interval for 95% confidence level (n = 3); [3] Indicative values

Table S8. Results (mg kg^{-1}, n = 3) for the determination of several REEs in NCS ZC 73006 Soil Reference Material by ICP–MS

Element	Certified Value ± U [1]	Found Value ± C.I. [2]	Recovery ± C.I. (%)
La	47 ± 2	38 ± 3	81 ± 8
Ce	93 ± 4	79 ± 6	85 ± 8
Pr	10.3 ± 0.8	8.8 ± 0.7	85 ± 8
Nd	41 ± 2	35 ± 3	85 ± 9
Sm	7.8 ± 0.3	6.8 ± 0.5	87 ± 7
Eu	1.56 ± 0.06	1.35 ± 0.10	87 ± 7
Gd	6.8 ± 0.5	6.9 ± 0.5	101 ± 7
Tb	1.08 ± 0.07	0.88 ± 0.06	81 ± 7
Dy	6.2 ± 0.4	5.2 ± 0.3	84 ± 6
Ho	1.23 ± 0.07	1.02 ± 0.05	83 ± 5
Er	3.4 ± 0.2	3.6 ± 0.2	106 ± 6
Tm	0.53 ± 0.04	0.52 ± 0.02	98 ± 4
Yb	3.5 ± 0.2	3.0 ± 0.2	86 ± 7
Lu	0.54 ± 0.02	0.44 ± 0.02	81 ± 5

[1] U is the expanded uncertainty for 95% confidence level (k = 2); [2] C.I. is the confidence interval for 95% confidence level (n = 3).

6. Operating conditions for 761 Compact IC Metrohm ion chromatograph

Table S9. Operating conditions for 761 Compact IC Metrohm ion chromatograph for anions determination in water samples

Column	Ion exchange METROSEP A Supp 5 – 100 / 4 (100 mm x 4 mm), Max. pressure 15 MPa, particle size 5 µm
Guard column	METROSEP A Supp 5 Guard / 4 (50 mm x 4 mm), particle size 5 µm
Mobile phase	Na_2CO_3 (0.0027 mol L^{-1})–$NaHCO_3$(0.001 mol L^{-1}) buffer solution 0.7 mL min^{-1}
Detection	Conductivity detector with chemical suppression
Injection loop volume	20 µL
Quantification	Peak area
Retention time	F^- - 2.548 min,<br>Cl^-, - 3.916 min<br>NO_3^- - 7.048 min<br>PO_4^{3-}- 8.693 min<br>SO_4^{2-} - 10.541 min

7. Limits of detection for anions determination in water by ion chromatography

Table S10. Limits of detection for anions determination by ion chromatography

Anion	LOD (mg L^{-1})
Cl^-	0.004
NO_3^-	0.100
SO_4^{2-}	0.010
NH_4^+	0.010
F^-	0.010
PO_4^{3-}	0.050

8. Relationship between major ions in water samples collected from Cloșani Cave considering all samples

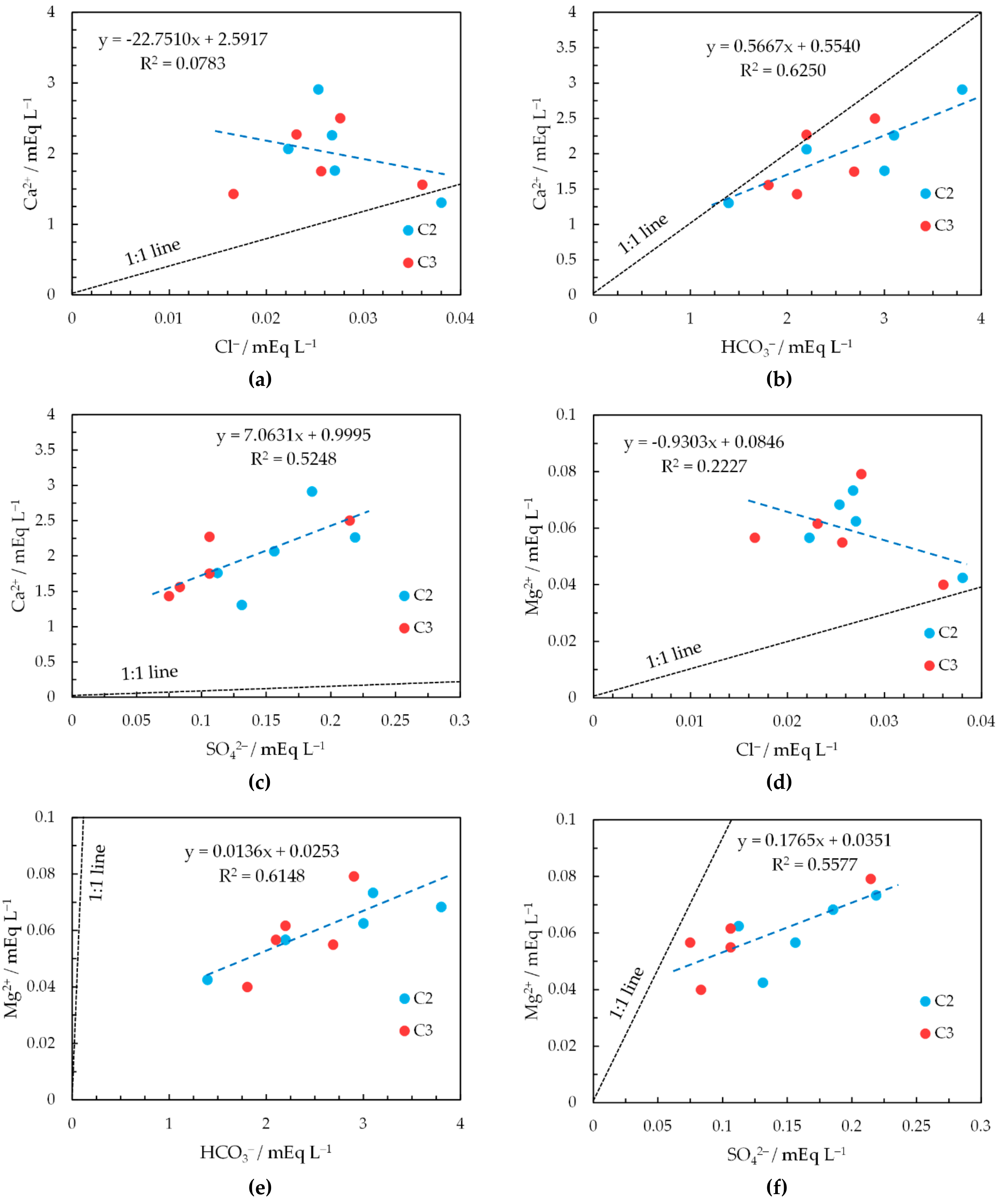

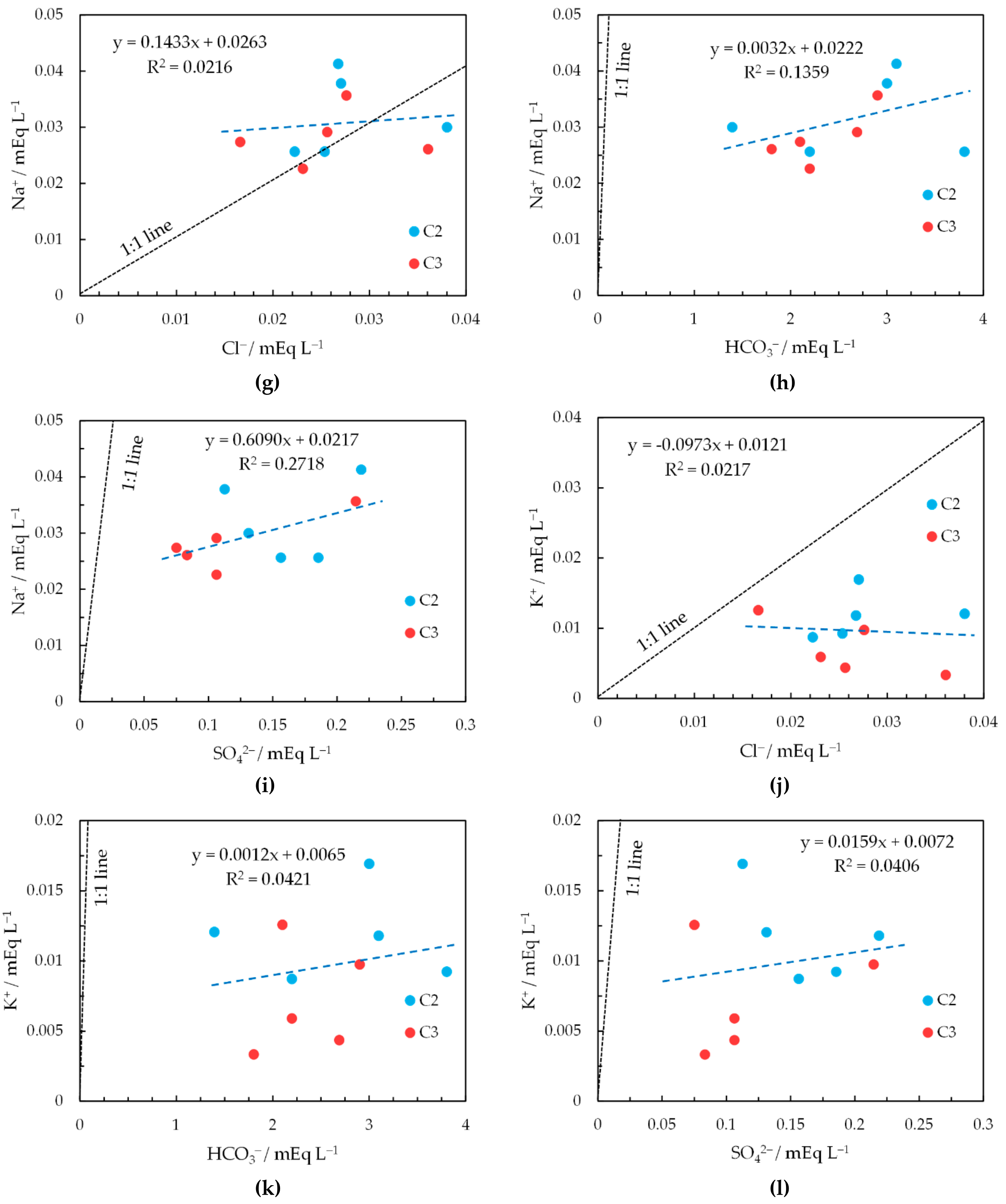

Figure S1: Relationship between major ions in water samples collected from Cloșani Cave considering all samples (a) Ca^{2+} – Cl^-; (b) Ca^{2+} – HCO_3^-; (c) Ca^{2+} – SO_4^{2-}; (d) Mg^{2+} – Cl^-; (e) Mg^{2+} – HCO_3^-; (f) Mg^{2+} – SO_4^{2-}; (g) Na^+ – Cl^-; (h) Na^+ – HCO_3^-; (i) Na^+ – SO_4^{2-}; (j) K^+ – Cl^-; (k) K^+ – HCO_3^-; (l) K^+ – SO_4^{2-}. C2 and C3 – sampling points.

9. Relationship between major ions in water samples collected from Cloșani Cave after the elimination of outliers caused by the seasonal variability

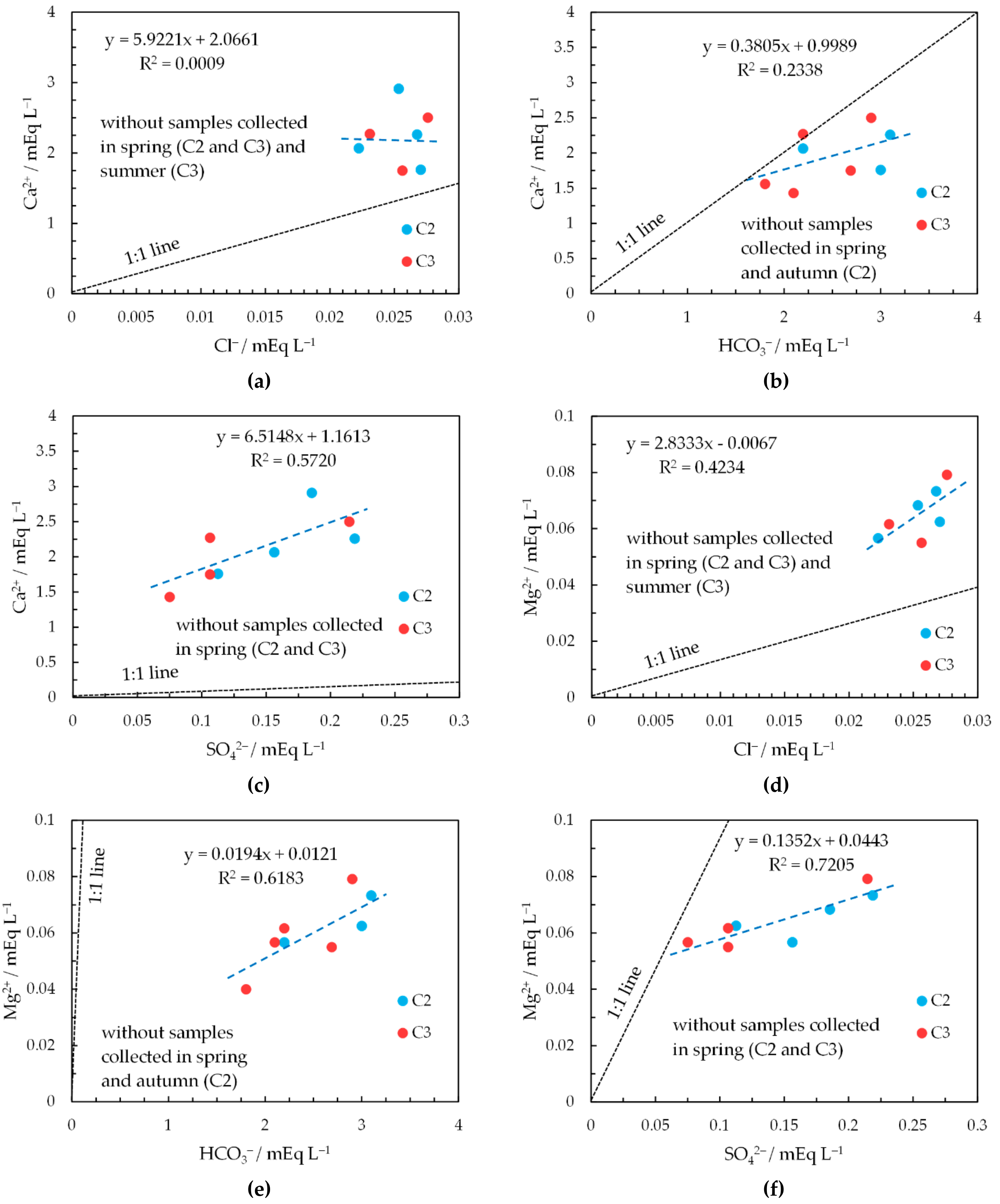

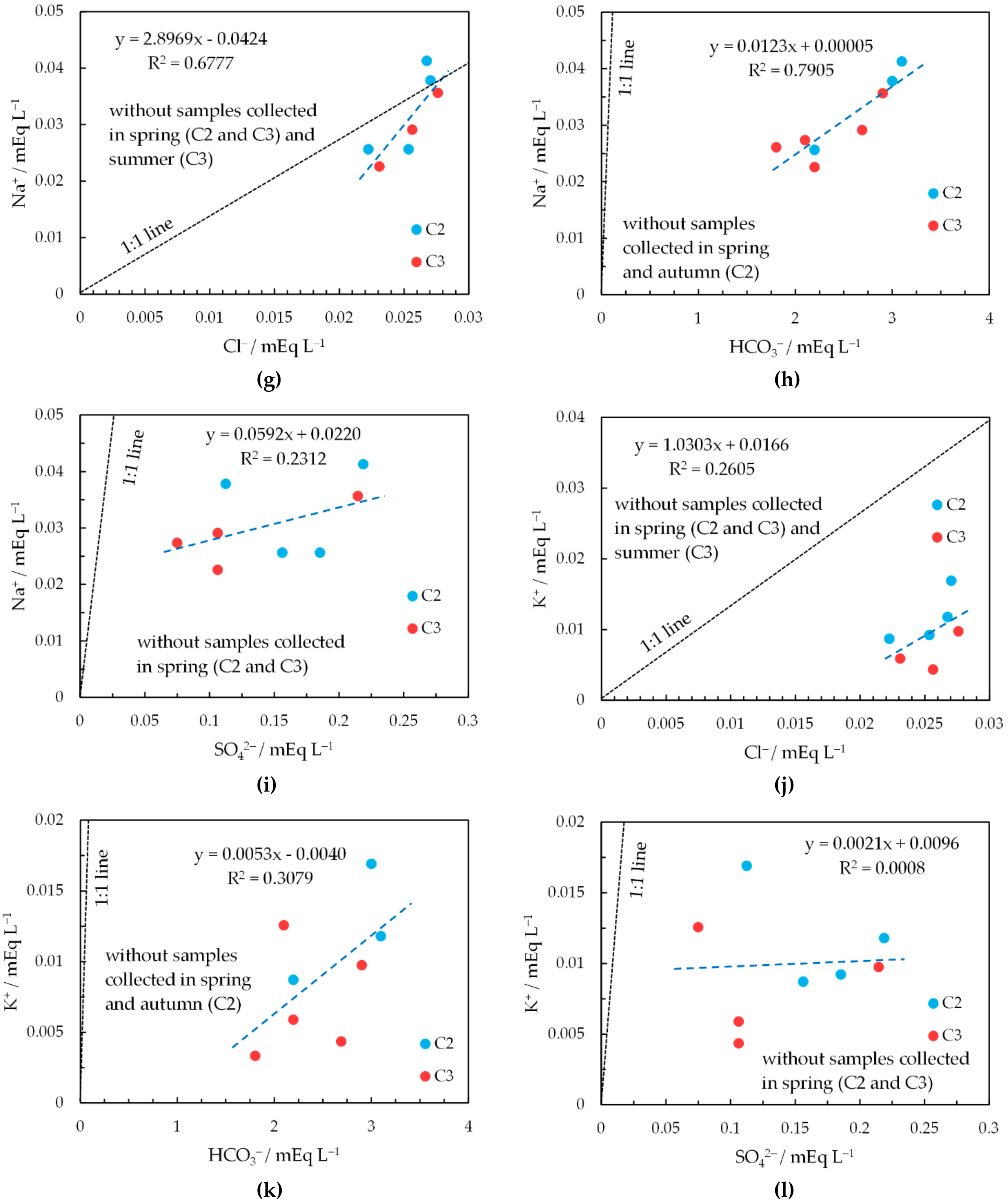

Figure S2: Relationship between major ions in water samples collected from Cloșani Cave after the elimination of outliers caused by the seasonal variability (a) Ca^{2+} – Cl^-; (b) Ca^{2+} – HCO_3^-; (c) Ca^{2+} – SO_4^{2-}; (d) Mg^{2+} – Cl^-; (e) Mg^{2+} – HCO_3^-; (f) Mg^{2+} – SO_4^{2-}; (g) Na^+ – Cl^-; (h) Na^+ – HCO_3^-; (i) Na^+ – SO_4^{2-}; (j) K^+ – Cl^-; (k) K^+ – HCO_3^-; (l) K^+ – SO_4^{2-}. C2 and C3 – sampling points

Author Contributions: Investigation, data curation, A.I.T.; data curation, writing - original draft preparation, E.A.L.; investigation, S.C.; funding acquisition, project administration, resources, supervision, writing—review and editing, O.T.M.; investigation, data curation, validation, M.S.; investigation, methodology, O.C.; formal analysis, D.C.; investigation, data curation, methodology, S.B.A.; investigation, data curation, methodology, C.T.; formal analysis, visualization, writing - original draft preparation, E.C.; conceptualization, supervision, writing—review and editing, T.F. All authors have read and agreed to the published version of the manuscript.

Funding: This research was supported by a grant of the Ministry of Research, Innovation and Digitization, CNCS/CCCDI –UEFISCDI, project number 2/2019 (DARKFOOD), within PNCDI III. The APC was funded by the Ministry of Research, Innovation and Digitization, CNCS/CCCDI – UEFISCDI, project number 2/2019 (DARKFOOD), within PNCDI III. S.C. acknowledge the support of the EEA Financial Mechanism 2014-2021, through the Grant SEE 126/2019 (KARSTHIVES) (Contract # 3/2019).

Institutional Review Board Statement: Not applicable.

Informed Consent Statement: Not applicable.
Data Availability Statement: The data presented in this study is available on request from the corresponding author.
Acknowledgments: The authors would like to thank Ionut, Cornel Mirea, Ruxandra Năstase-Bucur and Cristian Sitar for their help with the samples collection.

Conflicts of Interest: The authors declare no conflict of interest. The funders had no role in the design of the study; in the collection, analyses, or interpretation of data; in the writing of the manuscript, or in the decision to publish the results.

Sample Availability: CRM samples are available from the manufacturer, while cave samples from the authors.
